# Comparative genome analyses of four rice-infecting *Rhizoctonia solani* isolates reveal extensive enrichment of homogalacturonan modification genes

**DOI:** 10.1186/s12864-021-07549-7

**Published:** 2021-04-07

**Authors:** Da-Young Lee, Jongbum Jeon, Ki-Tae Kim, Kyeongchae Cheong, Hyeunjeong Song, Gobong Choi, Jaeho Ko, Stephen O. Opiyo, James C. Correll, Shimin Zuo, Sheshu Madhav, Guo-Liang Wang, Yong-Hwan Lee

**Affiliations:** 1 Department of Plant Pathology, The Ohio State University, Columbus, OH 43210 USA; 2 Fungal Bioinformatics Laboratory, Seoul National University, Seoul, 08826 South Korea; 3 Interdisciplinary Program in Agricultural Genomics, Seoul National University, Seoul, 08826 South Korea; 4 Department of Agricultural Life Science, Sunchon National University, Suncheon, 57922 South Korea; 5 Department of Agricultural Biotechnology, Seoul National University, Seoul, 08826 South Korea; 6 Ohio Agricultural Research and Development Center (OARDC) Molecular & Cellular Imaging Center (MCIC)-Columbus, The Ohio State University, Columbus, OH 43210 USA; 7 Department of Entomology & Plant Pathology, University of Arkansas, Fayetteville, AK 72701 USA; 8 Key Laboratory of Plant Functional Genomics of the Ministry of Education/ Jiangsu Key Laboratory of Crop Genomics and Molecular Breeding, Agricultural College of Yangzhou University, Yangzhou, China; 9 Indian Council of Agricultural Research-Indian Institute of Rice Research (ICAR-IIRR), Hyderabad, 500030 Telangana India; 10 Center for Fungal Genetic Resources, Plant Immunity Research Center, Plant Genomics and Breeding Institute, and Research Institute of Agriculture and Life Sciences, Seoul National University, 08826 Seoul, South Korea

**Keywords:** *Rhizoctonia solani* AG1-IA, Rice sheath blight, Plant cell wall degrading enzymes, Homogalacturonan/pectin modification genes

## Abstract

**Background:**

Plant pathogenic isolates of *Rhizoctonia solani* anastomosis group 1-intraspecific group IA (AG1-IA) infect a wide range of crops causing diseases such as rice sheath blight (ShB). ShB has become a serious disease in rice production worldwide. Additional genome sequences of the rice-infecting *R. solani* isolates from different geographical regions will facilitate the identification of important pathogenicity-related genes in the fungus.

**Results:**

Rice-infecting *R. solani* isolates B2 (USA), ADB (India), WGL (India), and YN-7 (China) were selected for whole-genome sequencing. Single-Molecule Real-Time (SMRT) and Illumina sequencing were used for de novo sequencing of the B2 genome. The genomes of the other three isolates were then sequenced with Illumina technology and assembled using the B2 genome as a reference. The four genomes ranged from 38.9 to 45.0 Mbp in size, contained 9715 to 11,505 protein-coding genes, and shared 5812 conserved orthogroups. The proportion of transposable elements (TEs) and average length of TE sequences in the B2 genome was nearly 3 times and 2 times greater, respectively, than those of ADB, WGL and YN-7. Although 818 to 888 putative secreted proteins were identified in the four isolates, only 30% of them were predicted to be small secreted proteins, which is a smaller proportion than what is usually found in the genomes of cereal necrotrophic fungi. Despite a lack of putative secondary metabolite biosynthesis gene clusters, the rice-infecting *R. solani* genomes were predicted to contain the most carbohydrate-active enzyme (CAZyme) genes among all 27 fungal genomes used in the comparative analysis. Specifically, extensive enrichment of pectin/homogalacturonan modification genes were found in all four rice-infecting *R. solani* genomes.

**Conclusion:**

Four *R. solani* genomes were sequenced, annotated, and compared to other fungal genomes to identify distinctive genomic features that may contribute to the pathogenicity of rice-infecting *R. solani*. Our analyses provided evidence that genomic conservation of *R. solani* genomes among neighboring AGs was more diversified than among AG1-IA isolates and the presence of numerous predicted pectin modification genes in the rice-infecting *R. solani* genomes that may contribute to the wide host range and virulence of this necrotrophic fungal pathogen.

**Supplementary Information:**

The online version contains supplementary material available at 10.1186/s12864-021-07549-7.

## Background

First reported in Japan in 1910 [1, 2], rice sheath blight (ShB) is one of the most devastating fungal diseases threatening rice production worldwide [3–5]. The causal agent of ShB is the soil-borne, necrotrophic fungi *Rhizoctonia solani* Kühn [teleomorph: *Thanatephorus cucumeris* (A.B. Frank) Donk], which belongs to the division Basidiomycota and subdivision Agaricomycotina. *R. solani* is a species complex that is classified into 13 anastomosis groups (AGs) based on the ability of genetically similar isolates to undergo hyphal fusion (anastomosis) [6–8]. Isolates in each AG are further categorized into intraspecific groups (ISGs) based on differences in host range, pathogenicity, cultural morphology, and biochemical characteristics [6, 8–10]. For instance, isolates of ISG IA belonging to AG1 (AG1-IA) can infect members of the Poaceae family including rice, maize, and turfgrass [11–13] to cause ShB, banded leaf sheath blight, and brown patch disease, respectively [3, 6, 14, 15]. Multiple studies have used genomic [13, 16–23], transcriptomic [16, 24] and proteomic approaches [25, 26] to examine the molecular basis for *R. solani* pathogenesis. A 36.94-Mbp draft genome sequence for *R. solani* AG1-IA isolated from infected rice in South China was assembled into 2648 scaffolds (for an N_50_ scaffold size of 474.5 Kb) in which 6156 genes were annotated [16]. However, more high-quality genome sequences from multiple rice-infecting isolates are needed to robustly identify conserved genomic signatures of rice-infecting *R. solani* AG1-IA.

Pathogenic fungi secrete various proteins to promote successful infection by suppressing host defenses and/or manipulating the physiology of host cells [27, 28]. Accordingly, this suite of proteins determines both the lifestyle and host ranges of these fungi [27]. A subset of these proteins is cysteine-rich, small (≤ 300 amino acids) secreted proteins (SSPs) called effectors [29, 30] that can form disulfide bridges to stabilize their tertiary structure, making them more resistant to degradation [31–33]. Plant pathogenic fungi also secrete carbohydrate-active enzymes (CAZymes) that allow them to breach the plant cell wall and enter their hosts [34–36]. These CAZymes are categorized into modules: glycoside hydrolases (GHs), carbohydrate esterases (CEs), auxiliary activities (AAs), glycosyltransferases (GTs), polysaccharide lyases (PLs), and (non-catalytic) carbohydrate-binding modules (CBMs) [36, 37].

This study aimed to sequence four genomes of *R. solani* isolated from ShB-infected rice and conducted comparative genome analyses amongst them (*R. solani* AG1-IA) as well as to 4 *R. solani* genomes belonging to AGs aside from AG1-IA (AG1-IB, AG2, AG3 and AG8). We hypothesized that rice-infecting *R. solani* would possess a large arsenal of cell wall-degrading genes to support its necrotrophic lifestyle and host range. To test this hypothesis, we assembled high-quality genome sequences for four rice-infecting *R. solani* strains that were isolated from rice grown in diverse geographic regions of the world (USA, China, and India) and compared them to publicly available genomes belonging to *R. solani* AG1-IA, AG2-IB, AG2-2IIIB, AG3, and AG8 [13, 16, 17, 19, 20]. We also selected representative genomes encompassing different nutritional lifestyles and hosts from Basidiomycota (9 genomes) and Ascomycota (9 genomes) into our comparative analyses. In this study, pairwise whole-genome alignments suggest that macrosynteny exists among the rice-infecting *R. solani* genomes. This phylogenetic proximity is supported by a phylogenetic tree constructed using the maximum likelihood method as well as the existence of a larger set of core-orthogroups among rice-infecting AG1-IA genomes (5812 orthogroups) compared to core orthogroups of *R. solani* genomes from diverse *R. solani* AGs (3635 orthogroups). Comparative genome analyses also revealed that rice-infecting *R. solani* have a smaller set of SSPs compared to biotrophs and other necrotrophs (cereal). Conversely, rice-infecting *R. solani* genomes code for the highest number of CAZymes, which are predicted to be involved in plant cell wall modification and degradation. Specifically, all *R. solani* genomes used in this study, regardless of AG, were highly enriched in pectin-degrading genes, containing even more than the well-known pectin-degrading, necrotrophic fungus *Verticillium dahliae*. The high-quality genome sequencing data and comparative genomic results from this study are useful resources for functional analysis of pathogenicity genes in this important fungal pathogen of rice.

## Results

### High-quality genome sequences for rice-infecting *R. solani* AG1-IA isolates

Four rice-infecting *R. solani* isolates were collected from rice grown in USA (B2), India (ADB, WGL), and China (YN-7) (Table 1). De novo sequencing of the B2 genome was achieved using a Single-Molecule Real-Time (SMRT; Pacific Biosciences); the WGL, ADB, and YN-7 genomes were sequenced using Illumina technology and subsequently assembled using the B2 genome as reference (Additional file 1: Fig. S1). B2 had the largest genome of the four isolates (45.01 Mbp; 96 scaffolds), while YN-7 had the smallest (38.92 Mbp; 413 scaffolds). ADB (39.90 Mbp; 811 scaffolds) and WGL (39.98 Mbp; 724 scaffolds) were intermediate in size. Accordingly, B2 had the highest number of protein-coding genes (11,505 genes), followed by WGL (10,044 genes), ADB (10,010 genes), and YN-7 (9715 genes). Despite these slight variations in genome size and gene numbers, all four genomes had similar total GC contents (47.3 to 47.7%). Based on scaffold numbers, which ranged from 96 to 811, and N_50_ values, which ranged from 1.22 Mbp to 1.56 Mbp, the newly sequenced *R. solani* AG1-IA B2 genome is of higher quality than the previously sequenced *R. solani* AG1-IA genome (2648 scaffolds; 36.94 Mbp) [16]. Thus, all comparative genomic analyses hereafter used the B2 genome as the representative for rice-infecting *R. solani* AG1-IA isolates.

**Table 1:** Genome statistics of the four rice-infecting *Rhizoctonia solani* AG1-IA (B2, ADB, WGL and YN-7), AG1-IB, AG2-2IIIB, AG3 and AG8 isolates

***Rhizoctoniasolani*** AG-ISG	Isolate	Host	Origin ofIsolate	Sequencingmethod	Genomesize (Mbp)	Scaffoldnumber	ScaffoldN_**50**_ (bp)	G-Ccontent(%)	Proteinnumber	Reference
**AG1-IA**	B2	Rice	USA (Arkansas)	PacBio de novo	45.0095	96	1,561,158	47.32	11,505	This study
	ADB	Rice	India	Reference-based Illumina	39.9044	811	1,218,423	47.53	10,010	
	WGL	Rice	India	Reference-based Illumina	39.9757	724	1,240,390	47.54	10,044	
	YN-7	Rice	China	Reference-based Illumina	38.9167	413	1,349,706	47.71	9722	
**AG1-1A**		Rice	China	Illumina GA II	36.9381	2648	474,500	47.6	10,489	[16]
**AG1-1B**	7–3-14	Lettuce	Germany	GenomeSequencer (GS) FLX	48.6737	23,356	N/A	48.1	12,268	[17]
**AG2-2IIIB**	BBA69670	Sugar beet	Germany	Illumina MiSeq	56.0285	2065	81,152	35.88	11,897	[19]
**AG3**	Rhs1AP	Potato	USA (Maine)	Sanger and GS-FLX 454	51.7059	326	N/A	48.4	12,726	[20]
**AG8**	WAC10335	Lupin	Australia	Illumina	39.8229	857	160,500	48.7	13,952	[13]

### Phylogenetic proximity of *R. solani* isolates

**Fig. 1:**
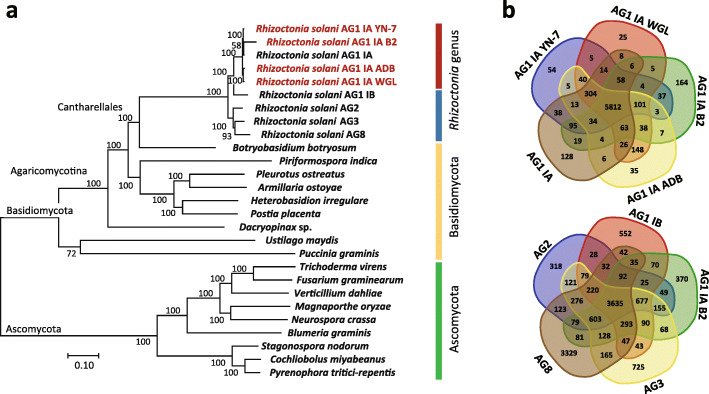
Evolutionary closeness of the genomes of rice-infecting *R. solani* AG1-IA and to that of the selected fungal outgroups used in this study. **a** Single-copy orthogroup, maximum likelihood-based phylogenetic tree illustrating the evolutionary proximity of *R. solani* isolates relative other members of the Basidiomycota and Ascomycota. **b** Intra- and inter- anastomosis group comparisons of orthogroups shared among the genomes of *R. solani* depicted in Venn diagrams

To evaluate the phylogenetic relationships of all 27 fungal genomes used in this study (Additional file 2: Table S1), we constructed a maximum likelihood-based phylogenetic tree using single-copy orthogroups (Fig. 1a). We confirmed that the four rice-infecting *R. solani* isolates were most phylogenetically related to each other and to the previously sequenced Chinese *R. solani* isolate, followed by AG1-IB, and finally the remaining *R. solani* AG isolates used in this study. In addition, comparison of the *R. solani* genomes indicated that the rice-infecting *R. solani* genomes share 5812 orthogroups of protein-coding genes while the genomes of different AG-ISG groups (AG1-IA B2, AG1-IB, AG2-2IIIB, AG3, and AG8) share only 3635 orthogroups (Fig. 1b). Of these orthogroups, 25 to 164 were specific to the genomes of rice-infecting *R. solani* AG1-IA while 318 to 3329 are AG-ISG specific.

### Genome synteny between *R. solani* isolates

**Fig. 2:**
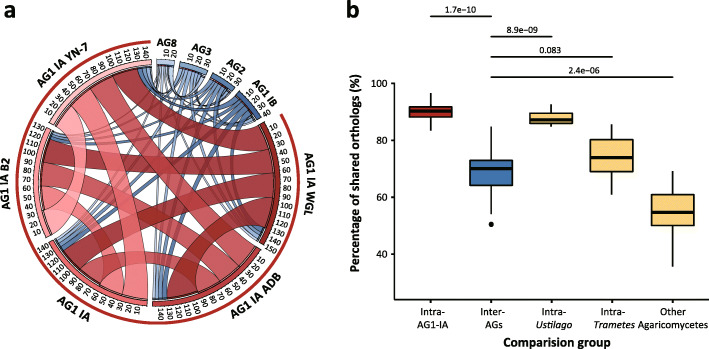
Genomic level synteny between *R. solani* anastomosis groups and proteome-level conservation of genes among and between the selected comparison groups. **a** Circos plot depicting the syntenic region size of all *R. solani* genomes used in this study. The outer block represents accumulated syntenic region size in Mbp calculated by PROmer. Red and blue blocks and ribbons represent AG1 and the rest of the anastomosis groups, respectively. **b** Comparison of the protein-coding gene proximity of five closely-related groups. Each consists of protein-coding genes of intra-AG-IA (rice-infecting *R. solani* AG1-IA), inter-AGs (AG1-IA B2, AG1-IB, AG2, AG3 and AG8), Basidiomycetes (*Piriformospora indica*, *Pleurotus ostreatus*, *Armillaria ostoyae*, *Heterobasidion irregulare*, *Dacryopinax* sp.), *Ustilago* and *Trametes*. The asterisks represent significant differences in distribution according to the t-test (*P* over 0.05, ∗∗∗*P* ≤ 0.001)

To determine the synteny between *R. solani* genomes, we performed pairwise whole-genome alignments using PROmer [38], a script-based pipeline to align multiple, divergent sequences and identify similar genomic regions based on the translation of all six reading frames. Large syntenic size (Fig. 2a) and diagonal dot plots (Additional file 3: Fig. S2) suggested that the five rice-infecting *R. solani* genomes (the four from this study and the previously sequenced Chinese isolate) share a high degree of genome conservation, ranging from 66 to 70.9% (Additional file 4: Table S2) when the B2 genome was used as reference for comparison. However, the degree of genome conservation between the B2 genome and those of different *R. solani* AGs (AG1-IB, AG2-2IIIB, AG3, and AG8) dramatically decreases to 3.3 to 9.6%, suggesting that the AG1-IA genome is quite divergent from other AGs.

### Protein-coding gene conservation among *R. solani* isolates

To determine the degree of proteome similarity in AGs, we performed pairwise ortholog clustering to compare each protein sequence of protein-coding genes. The number of shared orthologs ranged from 8798 to 9723 in the protein-coding genes of *R. solani* AG1–1A isolates (Additional file 5: Table S3). The average proteome similarity of intra-AGs of *R. solani* was 89.69%. In contrast, the protein-coding gene similarity of inter-AGs of *R. solani* was more diversified, wherein the percentage of shared predicted proteomes in inter-AGs was averagely 68.36%. Moreover, in order to determine protein-coding gene similarity of genomes belonging to Basidiomycota, we added four *Ustilago* and four *Trametes* genomes along with five Agaricomycetes genomes used for phylogenetic analyses (Additional file 6: Table S4). *R. solani* genomes of inter-AGs (compared with B2 strain) were larger than that of different Basidiomycota genus-group (54.53%). On the contrary, intra-genus group under Basidiomycota showed high protein-coding gene similarity compared to inter-AG (*Ustilago* intra-genus; 88.08%, *Trametes* intra-genus; 83.41%) (Fig. 2b). In comparison of single copy orthologs, discrepancy of inter AGs were increased (intra-AG1 IA; 63.89%, inter-AGs; 33.27%).

### Transposable element profiles of the rice-infecting *R. solani* isolates

Transposable elements (TEs), such as class I retrotransposons and class II DNA transposons, can create temporary or permanent genomic rearrangements and modifications [39], and the abundance and frequency of these genetic elements can significantly influence the size of eukaryotic genomes [40]. To define the repetitive element profiles for the rice-infecting *R. solani* genomes, we analyzed the type and proportion of repetitive elements in each newly sequenced genome. The B2 genome contains the largest proportion of TEs (26.74%) compared to three other *R. solani* AG1-IA genomes; ADB (8.89%), WGL (9.16%), and YN-7 (6.18%), respectively (Additional file 7: Table S5). The number of total TEs for the ADB and WGL genomes are comparable (ADB: 10,421, WGL: 10,248), yet less than that of the B2 genome (17,123) and more than that of YN-7 (8030). Specifically, the B2 genome contains the highest proportion of DNA transposons, Long Terminal Repeats (LTRs) and Long Interspersed Nuclear Elements (LINEs) among the *R. solani* AG1-IA genomes, wherein the proportion of LTRs in B2 (20.14%) was identified to be more than 3 times of that of ADB (5.47%), WGL (5.65%) and YN-7 (3.84%). However, all four newly sequenced AG1-IA genomes as well as the previously sequenced AG1-IA genome possess lower numbers of TEs compared to AG1-IB, AG2-2IIIB, AG3 and AG8. In terms of average length of repetitive sequences, the PacBio-sequenced B2 genome contains approximately twice as long (797.4 bp) compared to the Illumina sequenced ADB (370 bp), WGL (385.6 bp), YN-7 (345.2 bp) in this study and other previously sequenced genomes of AG1-IA, AG1-IB, AG2-2IIIB, AG3 and AG8.

### Predicted secretome of rice-infecting *R. solani* isolates

**Fig. 3:**
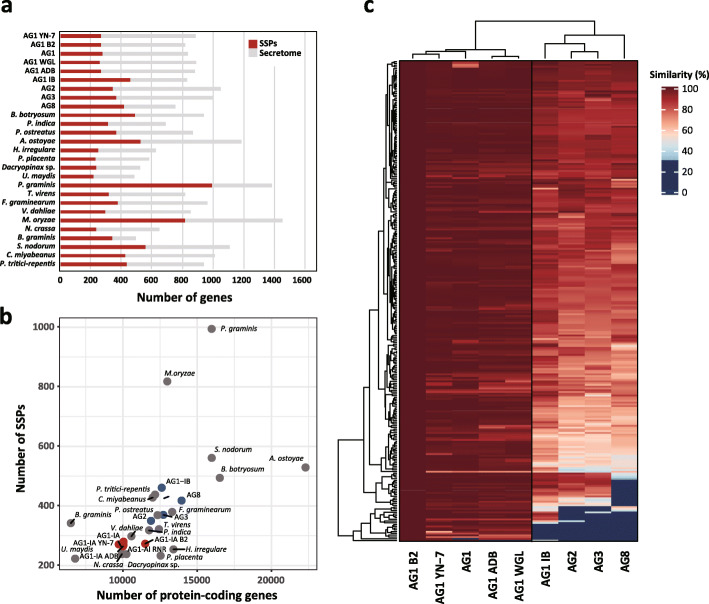
Distribution of small secreted proteins in *R. solani* isolates and other fungal species. **a** The number of small secreted proteins in the total secretome of each fungal genome. Gray and red bars represent the size of secretome and the number of small secreted proteins, respectively. **b** The number of SSPs in relation to the number of total protein-coding genes. Red, blue, gray dots represent genomes belonging to intra-AGs, inter-AGs, and other fungal species. **c** The heatmap shows the conservation of 272 SSPs in B2 against the other *R. solani* genome sequences. Exonerate 2.4.0 was utilized to perform protein to genome sequence alignments of the SSPs

The putative secretomes of the rice-infecting *R. solani* isolates were analyzed, and 818 to 888 predicted secreted protein genes were identified (Fig. 3a, Additional file 8: Table S6). This suggests that rice-infecting *R. solani* isolates have a secretome that is intermediate in size smaller than those of necrotrophic ascomycetes (cereal) but larger than those of brown-rot fungi *Postia placenta* and *Dacryopinax* sp. as well as biotrophs *Ustilago maydis* and *Blumeria graminis*. The number of small secreted proteins (SSPs; putative effectors) in the four genomes ranges from 263 to 279, accounting for 30–33% of each isolate’s individual predicted secretome. We identified 367 *R. solani* specific orthogroups of SSPs. Among these specific SSPs, 12 (AG1-IB) to 105 (AG8) AG-specific SSPs were identified through ortholog comparison analysis of putative SSP gene sets (Additional File 9: Table S7), and the greatest number of specific SSPs were identified in AG8. We also observed that *Rhizoctonia* AG1-IA genomes have relatively small predicted protein-coding genes and SSPs are shown to be necrotrophic fungal groups (Fig. 3b).

Furthermore, using the 272 SSPs identified in B2, we performed alignment of their protein sequence to the genomes of *R. solani* AG1-IA, AG1-IB, AG2-2IIIB, AG3, and AG8. The B2 SSPs possess a high degree of homology amongst rice-infecting AG1-IA genomes, whereas they showed decreased homology to genomes of other *R. solani* AGs (Fig. 3c, Additional file 10: Table S8).

### Predicted CAZyme genes of the rice infecting *R. solani* isolates

**Fig. 4:**
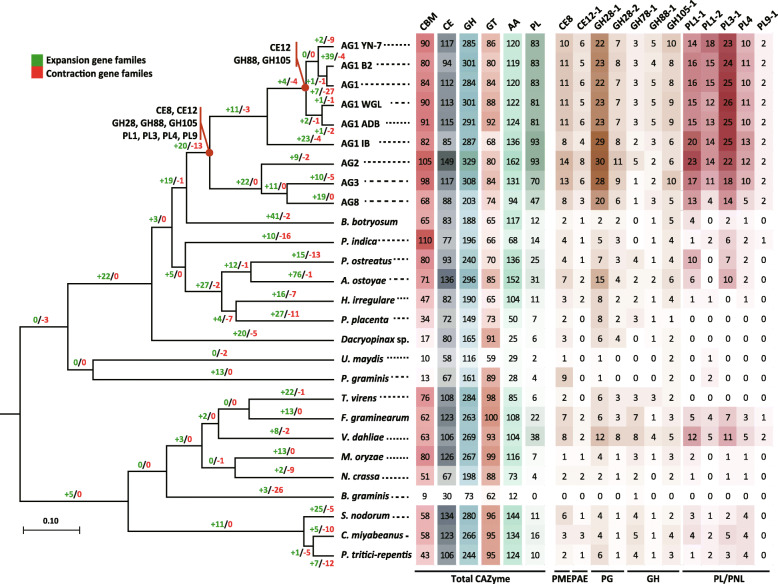
Distribution of gene families in rice-infecting *R. solani* AG1-IA isolates and the fungal outgroups used in this study. Phylogenetic tree with information of contracted and expanded gene families. Abundance of genes in carbohydrate-binding module (CBM), glycoside hydrolase (GH), carbohydrate esterase (CE), glycosyltransferase (GT), polysaccharide lyase (PL) and auxillary activity (AA) families. Expansion and contraction of enriched pectin lyase and pectate lyase (PL/PNL: PL1–1 (EC 4.2.2.2), PL1–2 (EC 4.2.2.10), PL3–1 (EC 4.2.2.2), PL4, PL9–1 (EC 4.2.2.2)), polygalacturonase (PG: GH28–1 (EC 3.2.1.15), GH28–2 (EC 3.2.1.67)), pectin methylesterase (PME: CE8) pectin acetylesterase (PAE: CE12–1 (EC 3.1.-)), and other GHs (GH105–1 (EC 3.2.1.172), GH88–1 (EC 3.2.1.-), GH78–1 (EC 3.2.1.40)) in all 27 fungal genomes used in this study indicated. Red circle indicates the gain EC in *R. solani* monophyletic

We identified cell wall degrading enzymes by searching for each of the different CAZyme gene families across all 27 fungal genomes (Fig. 4). These fungal genomes were then categorized into 11 groups considering their nutritional lifestyle and type of host. A chi-square test of proportions was then used to determine whether gene frequency variations between genomes of each grouping were significant (Additional file 11: Table S9 and Additional file 12: Table S10). Our analyses indicated that there was significant variation across all 11 groups for all CAZyme gene families except GTs. Rice-infecting *R. solani* genomes had the highest enrichment of CAZyme genes (725 genes) while the genomes of other *R. solani* AGs, necrotrophs (cereal and dicot) showed only moderate enrichment for these genes. In contrast, biotrophs and brown rot genomes contain a relatively low number of CAZymes compared to rice-infecting *R. solani* genomes.

### Lignocellulose-degrading genes in rice-infecting *R. solani* isolates

To ascertain whether rice-infecting *R. solani* isolates can degrade lignocellulose in a similar fashion to fungi in the subdivision Agaricomycotina, we specifically searched for AA family-encoding genes (Additional file 13: Table S11). Genomes of white-rot fungi and other *R. solani* AGs had the highest total number of CAZyme genes (131 genes), followed by necrotrophs (cereal) (128 genes) and rice-infecting *R. solani* (121 genes). In contrast, brown-rot and biotroph genomes completely lacked these lignin depolymerization genes. We also examined the genomes to identify genes belonging to individual AA subfamilies. Genes belonging to the AA1 subfamily (EC 1.10.3.2) were most abundant in white-rot fungi but could also be found in rice-infecting *R. solani* isolates, hemibiotrophs, and necrotrophs (cereal); however, the presence of these AA1 genes was significantly lower in brown-rot fungi and biotrophs. In addition, while brown-rot and rice-infecting *R. solani* genomes did not contain any representatives of the AA2 subfamily, these genes were present in the genomes of necrotrophs (cereal) and white-rot fungi; the latter were highly enriched in manganese peroxidase (MnPs; 1.11.1.13) and versatile peroxidase (VPs; 1.11.1.16) genes. We also observed enrichment of AA8 subfamily genes in the genomes of rice-infecting *R. solani* isolates, while white-rot genomes had only 1 or 2 AA8 genes and cereal necrotroph genomes had none (except for *Septoria nodorum*, which had 1). Finally, the AA5 subfamily, which was absent in brown-rot fungi, was significantly enriched in other *R. solani* AGs and to a lesser extent in rice-infecting *R. solani* and white-rot fungi.

We assessed the abundance of cellulose-degrading genes across all genomes in a similar fashion (Additional file 13: Table S11). Endoglucanase genes (GH3) were equally abundant in rice-infecting *R. solani* isolates and necrotrophs (cereal). However, exo-1,3-β-glucanase (3.2.1.58, GH5), endo-1,6-β-D-glucanase (3.2.1.75, GH5), and cellulose 1,4-β-cellobiosidase (3.2.1.176, GH7) genes were enriched in rice-infecting *R. solani* isolates but absent in necrotrophic fungi (cereal). Strikingly, rice-infecting *R. solani* genomes were highly enriched in genes encoding starch-degrading α-amylase (3.2.1.1, GH13). Moreover, genes encoding endo-1,4-β-D-glucanohydrolase (3.2.1.4, GH5), which causes endohydrolysis of (1 → 4)-β-D-glucosidic linkages in cellulose, lichenin, and cereal β-D-glucans, were enriched in rice-infecting *R. solani* and *S. nodorum*. Finally, genes encoding lytic polysaccharide monooxygenases (LPMOs) of the AA9 subfamily were significantly enriched in other *R. solani* AGs, though they were also present in rice-infecting *R. solani*. In contrast, biotrophs and brown-rot fungi had, at most, one AA9 gene.

### Pectin-degrading and modifying genes in rice-infecting *R. solani*

Extensive enrichment of genes belonging to the PL family was observed in both rice-infecting (82 genes) and other *R. solani* AGs (76 genes) (Additional file 13: Table S11). Otherwise, only necrotrophic (dicot) *V. dahliae* was predicted to have an enriched number of PL genes, though it only had approximately half as many PL genes as *R. solani*. In-depth analyses of the PL subfamilies revealed that genes encoding members such as pectate lyases (EC 4.2.2.2.2; PL1, 3, 9), pectin lyases (EC 4.2.2.10; PL1), and rhamnogalacturonan endolyases (EC 4.2.2.-, PL4) were significantly enriched in rice- and other *R. solani* AGs.

GH genes that encode pectin-degrading enzymes like unsaturated rhamnogalacturonyl hydrolase (EC 3.2.1.172, GH105), unsaturated β-glucuronyl hydrolase (3.2.1.-, GH88), and α-L-rhamnosidase (3.2.1.40, GH78) were analyzed in a similar fashion. GH28 genes were more enriched in rice-infecting *R. solani* than in necrotrophs (cereal) and *V. dahliae*. Specifically, 13 to 14 polygalacturonase (PGs) (EC 3.2.1.15, GH28) genes were found in AG1-IA genomes and other *R. solani* AG genomes while *V. dahliae* only had 2 PG genes. Exo-PGs (EC 3.2.1.67, GH28) were similarly abundant and enriched in *R. solani* and *V. dahliae*. Meanwhile, CE genes that encode pectin-modifying enzyme genes, including pectin methylesterases (PMEs; CE8) and pectin acetylesterases (CE12; EC 3.1.1.-), were most abundant in all *R. solani* genomes. All of the enriched genes belonging to the homogalacturonan modification genes (PAE, PME, PG, and PL/PNL) have largely expanded when the *R. solani* divergence occurred (Additional file 14: Fig. S3).

### Genes for monocot-specific cell wall degrading enzymes

To determine whether rice-infecting *R. solani* isolates have any CAZyme genes that allow them to infect their monocot host, we searched for CAZyme genes that degrade arabinoxylans, ferulic acids, and mixed linked glucans (MLGs), such as α-L-arabinofuranosidases (EC_3.2.1.55), feruloyl esterases (EC_3.1.1.73), and (1,3;1,4)-β-D-glucan endohydrolases/licheninase (EC 3.2.1.73), respectively (Additional file 15: Table S12). α-L-arabinofuranosidase genes were enriched in necrotrophs, including *R. solani* AG1-IA and other *R. solani* AGs, but not in white- and brown-rot fungi. However, while feruloyl esterase genes were enriched in necrotrophs (cereal), symbionts, and hemibiotrophs, none were identified in rice-infecting *R. solani*. While the raw data suggested that there was an enrichment in genes encoding (1,3;1,4)-β-D-glucan endohydrolases/licheninases in rice-infecting *R. solani* genomes, our chi-square test failed to reject the null hypothesis, so the proportion of (1,3;1,4)-β-D-glucan endohydrolases/licheninases genes across the different fungal genomes is likely similar.

### Prediction of secondary metabolite biosynthesis gene clusters

antiSMASH [41] was used to identify putative secondary metabolite biosynthesis gene clusters for polyketide synthase (PKS), terpene synthase (TS), non-ribosomal peptide synthetase (NRPS), and other accessory enzymes in rice-infecting *R. solani* isolates (Additional file 15: Table S12). However, none of the secondary metabolite biosynthesis gene clusters predicted for rice-infecting *R. solani* isolates or members of related AGs contained PKS genes. In contrast, there was an abundance of secondary metabolite-producing enzymes predicted for necrotrophic ascomycetes (cereal), including type 1 PKS, type 2 PKS, NRPS, and TS. Despite the low abundance and diversity of putative secondary metabolite biosynthesis gene clusters in *R. solani* genomes, *R. solani* AGs and necrotrophic ascomycetes (cereal) have similar levels of terpenes, which suggests that secondary metabolites may not be important for *R. solani* virulence.

## Discussion

### De novo and reference-based genome assemblies of rice-infecting *R. solani* isolates

Both SMRT and Illumina sequencing technologies were used in the de novo sequencing of the B2 genome, which allowed us to utilize (1) the ability of SMRT sequencing to generate long read lengths (5 to 20 kb) and precisely capture genomic regions containing repetitive elements and novel gene isoforms [42]. Utilizing these sequencing approaches facilitated assembly of the 45-Mbp B2 genome, which is much larger than a previously published 36.94-Mbp *R. solani* AG1-IA genome and has a relatively high proportion of repetitive sequences. In addition, it also provided an opportunity to accurately annotate the TE content of the B2 genome. Upon comparing to both newly and previously sequenced *R. solani* AG1-IA genomes as well as to genomes of other AGs, the B2 genome was found to possess the highest proportion of TEs and the longest average length of TEs. Thus, the higher quality B2 genome generated from the two sequencing methods allowed us to more accurately annotate the *R. solani* AG1-IA genomes for detailed comparative genome analyses of this important fungal pathogen.

### Genomic differences among *R. solani* isolates

In previous studies, *R. solani* isolates have been classified based on their ability to hyphal fuse or through sequence analysis of phylogenetic markers [9, 43, 44] but whole-genome comparisons were not available. Here, we compared five *R. solani* AG1-IA genomes and four neighboring AG representative genomes and found out that genomic drastically decreased as the comparisons were made from among AG1-IA genomes to among different AG group genomes. Moreover, the similarity of inter-AGs was lower than other Basidiomycota genus-groups. This phylogenomic result shows *R. solani* species complex reflects multi-species feature in genome contents.

### Small set of predicted putative effectors in rice-infecting *R. solani* isolates

It has been shown that necrotrophic fungi have fewer effectors than biotrophs [45]. Along this line, we found that the newly sequenced rice-infecting *R. solani* AG1-IA genomes had relatively small set of effectors among fungal genomes analyzed in this study. Previous reports suggest that the broad host range of *R. solani* is not dependent on the size of its secretome [46] but rather on the secretion of specific effectors than can infect a variety of different hosts, as is the case with necrotroph *Sclerotinia sclerotiorum* [47]. Furthermore, it has been reported that *Colletotrichum* pathogens from different clades have tailored suites of CAZymes that are specific to their individual host range and infection lifestyle [48]. We speculate that the relatively low number of SSPs (putative effectors) may be compensated by the diverse and large arsenal of CAZymes of rice-infecting *R. solani*, allowing them to be a competitive pathogen of broad host range. Additional bioinformatic and functional genomics analyses must be conducted in order to dissect the role of SSPs in rice-infecting *R. solani* genomes. However, we have provided evidence that the SSPs among the rice-infecting *R. solani* genomes share high homology and decrease homology of these SSPs among genomes of different *R. solani* AGs suggest that the SSPs of from *R. solani* species complex as a whole may be diverse that previously expected.

### Lignocellulose-degrading CAZyme genes in rice-infecting *R. solani* isolates

Essential for cell growth and differentiation, cell walls are primarily comprised of cellulose, hemicellulose, pectin, and lignin [49–53], which also provide plants with resistance to and protection from biotic and abiotic stresses [54–56]. Plant biomass-degrading fungi that inhabit diverse ecological niches such as forest litters, trees, crops, and grasses of the subdivision Agaricomycotina [34] are classified as either white- or brown-rot [57] based on their ability to degrade lignin. White-rot fungi use oxidative enzymes [58, 59] like glyoxal oxidases to efficiently depolymerize lignin and class II heme peroxidases of the AA2 subfamily, such as MnPs and VPs, to degrade the lignin matrix and expose the embedded cellulose [60, 61]. While we observed enrichment of glyoxal oxidase-encoding genes in rice-infecting *R. solani* genomes, we could not identify any peroxidase genes in the rice-infecting *R. solani* or brown-rot genomes. This apparent lack of peroxidases may explain why brown-rot fungi rapidly degrade cellulose but leave behind a chemically modified lignin matrix for long-term degradation by other microbes [62–64].

While a relationship between modes of wood decomposition and CAZyme families exists, some wood-decaying fungi cannot be strictly categorized as white- or brown-rot, suggesting that a continuum may exist between these two types of wood-decay fungi [57]. Rice-infecting *R. solani* isolates were enriched in lignocellulose-degrading CAZyme genes as well as strong cellulose-degrading enzymes, which are hallmarks of white- and brown-rot fungi, respectively. Hence, we hypothesize that *R. solani* may fall along the continuum between these two types of wood-decay fungi. Furthermore, the abundance of oxidoreductase and iron reductase genes in the *R. solani* genomes may indicate that these enzymes are involved in the production of hydroxyl radicals to drive lignocellulose attack, thereby working with crystalline cellulose-degrading LPMOs to expose the complex lignocellulose structure for further cell wall degradation by other CAZymes.

### Enrichment of pectin-degrading enzymes in rice-infecting *R. solani* isolates

Pectin is a structural heteropolysaccharide with a 1,4-α-D-galacturonic acid (GalA) backbone that contributes to the mechanical strength of plants [65–67] by forming a gel-like matrix that interacts with cellulose and hemicellulose in the primary plant cell wall [51, 68]. It is present in higher proportions in dicot (type I) cell walls than in monocot (type II) cell walls [50], and some reports suggest that dicot-specific fungal pathogens have higher amounts of pectin-degrading enzymes than monocot-specific pathogens [69, 70]. Pectin is classified based on the degree of methoxylation, methylesterification, and/or acetylation of its backbone [71, 72]. Homogalacturonan (HG), a polymer of 1,4-linked α-D-galactopyranosyluronic acid, exists in methylesterified or acetylated form in the primary cell walls of plants [65], and pectin methylesterases (PMEs; EC 3.1.1.11) and pectin acetylesterases (PAE; EC 3.1.1.6) catalyze the demethylesterification and deacetylation of HG, respectively [73–75]. This process yields substrates for PGs [76], pectin lyases (PNLs; EC 4.2.2.10), and pectate lyases (PLs; EC 4.2.2.2), which loosen the cell wall [77]. Demethylesterification of HGs can also lead to ‘egg-box formation’ and cell wall stiffening caused by the interaction of negatively-charged demethylesterified HG and divalent cations such as calcium ions [77], which may explain the stiff, hollow-textured stem phenotypes observed in ShB-susceptible rice cultivars but not in moderately ShB-resistant rice cultivars (Lee et al., unpublished data). The significant enrichment of PMEs, PAEs, PGs, PNLs, and PLs in *R. solani* genomes suggests that these pathogens may have evolved a diverse suite of pectin depolymerization enzymes that allow them to efficiently breach host cell walls. These expanded homogalacturonan modification genes are known to have the enzymatic activity of cell wall loosening roles in the infection process. Similarly, mutations in *Arabidopsis thaliana* pectin methylesterase 35 (PME35) lead to the suppression of HG demethylesterification and a concomitant increased stem deformation rate, supporting the essential role of pectin in maintaining the integrity of the plant cell wall and supporting the plant’s mechanical properties [65].

Gene family expansions and contractions are the signatures of an organism’s adaptation to new ecological niches [78], as exemplified by the presence of numerous pectin-degrading genes in rice-infecting *R. solani* and neighboring *R. solani* AGs. However, dicot-specific fungal pathogens may not necessarily have more specialized pectin-depolymerizing enzyme suites than monocot-specific pathogens, as genes encoding HG-modifying enzymes are more highly enriched in rice-infecting *R. solani* isolates than in the dicot-specific pathogen *V. dahliae*. This enrichment in pectin degrading genes indicates that *R. solani* can degrade a wide range of pectic substrates and polysaccharide linkages, allowing it to use multiple virulence mechanisms to invade a variety of hosts. Moreover, the large and diverse suite of pectin-degrading enzymes in rice-infecting *R. solani* isolates may not have evolved in response to the amount of pectin in host plant cell walls but rather as an efficient mechanism for loosening plant cell walls, breaking crosslinks with other cell wall components, and dissolving plant tissue.

### Monocot-specific degrading genes in rice-infecting *R. solani* isolates

Previous studies have shown a correlation between cell wall composition and host specificity, suggesting that plant pathogens produce host-specific cell wall degrading enzymes [79, 80]. Accordingly, monocot-specific fungal pathogens are adept at hydrolyzing monocot cell walls while dicot-specific fungal pathogens are better at degrading dicot cell walls [81]. Non-cellulosic polysaccharides such as arabinoxylans, MLGs, and hydroxycinnamates such as ferulic acids, which are enriched in grass cell walls but either limited or absent in dicot cell walls [50, 82–84], are degraded by α-L-arabinofuranosidases (EC 3.2.1.55, GH51) [81, 85], licheninases [86], and feruloyl esterases (EC 3.1.1.73) [34]. However, we did not observe significant differences in the number of monocot-specific cell wall degrading CAZyme genes present in the different rice-infecting *R. solani* genomes, which may indicate the broad host range of this necrotrophic fungus [13].

### Secondary metabolite biosynthesis clusters in *R. solani* isolates

Previous studies suggest the association of loss of secondary metabolite genes with biotrophy [87, 88]. However, most of the pathways for secondary metabolite synthesis in the biotrophic fungus *Cladosporium fulvum* were revealed to be cryptic [89]. Despite our results suggest that *R. solani* possess limited number of secondary metabolite biosynthesis clusters, further research on expression analyses of the putative secondary metabolite genes and those identified along with metabolite extraction and chromatography will be needed. These analyses will provide conclusive evidence about the extent of involvement of secondary metabolite genes in the lifestyle and pathogenicity of *R. solani*.

## Conclusion

In this study, we analyzed the cell wall degrading enzyme profiles of four newly sequenced rice-infecting *R. solani* genomes. Comparative analyses of these rice-infecting *R. solani* genomes can help identify cell wall degrading mechanisms, such as homogalacturonan modification, that are utilized by this necrotrophic, rice-infecting ShB pathogen. With more and more *R. solani* genomes are sequenced in the future, reclassification of this fungal pathogen should be discussed and implemented. Moreover, our findings, along with the high-quality genome sequences of rice-infecting isolates of *R. solani* AG1 IA, provide additional genomic resources that can be used to further our understanding of the pathobiology of this necrotrophic fungal pathogen.

## Methods

### Sources of *R. solani* AG1-IA isolates

Four *R. solani* AG1-IA isolates were collected from rice cultivars grown in the USA, India and China with famers’ permission. B2 was recovered from Jerry Bogard Farms, Stuttgart, Arkansas, USA. ADB and WGL were recovered from Srinivasa Rao Farms in Adilabad and Bose Reddy Farms in Warangal in Telangana State, India, respectively. YN-7 was recovered from Zongliang Chen Farms in Yangzhou, China.

### Fungal DNA extraction of *R. solani* isolates

Hyphal tip isolation and culture maintenance of *R. solani* isolates were conducted using Potato Dextrose Agar (PDA). *R. solani* hyphae-containing agar blocks were isolated from the actively growing mycelial portion of the fungus and cultured in the dark in liquid Potato Dextrose Broth (PDB) at 25 °C on an orbital shaker (150 rpm) for 4–5 days. Mycelia were filtered using sterile Miracloth (Millipore, Sigma, Burlington, MA, USA), rinsed with sterile distilled water, and frozen in liquid nitrogen. Genomic DNA (gDNA) was extracted using a DNeasy Plant Mini Kit (Qiagen, Valencia, CA, USA), and the resulting DNA pellet was resuspended in 10 mM Tris-HCl (pH 8.0) buffer. The quality of the isolated gDNA was assessed using agarose gel-based electrophoresis while the total DNA concentration was calculated based on UV-Vis measurements on a Nanodrop spectrophotometer. Isolated gDNAs were sent to the National Instrumentation Center for Environmental Management (NICEM), Seoul National University, Korea, for SMRT and Illumina-based sequencing.

### Genome sequencing, assembly, and annotation

A PacBio sequencing assemblage strategy was used to assemble the B2 genome. Raw PacBio RSII sequence reads were assembled and corrected using Canu v2.0 [90] and trimmed using Circlator v0.14.0 [91]. All resulting contigs were then joined before the Redundans assembly pipeline [92] and Pilon v1.22 [93] was used to improve the final draft genome.

For Illumina-based sequencing and assembly of the ADB, WGL, and YN-7 genomes, contigs were aligned to the PacBio RSII-sequenced B2 genome. As with B2, the Redundans assembly pipeline was used to process Illumina sequencing reads, but with an additional step: filtered Illumina reads by FASTQC v0.11.6 [94] were assembled using Jellyfish v1.1.5 [95]. Alignment and assembly were performed using SOAPdenovo2 [96] and Velvet v1.2.10 [97]. Gap-filling within scaffolds was achieved using GapCloser v1.12-r6 [98]. The resulting sequences were joined and corrected using Pilon v1.22. AUGUSTUS v3.2.2 [98] was used to make gene annotation predictions based on deposited six different protein-coding genes of *R. solani* in NCBI (AG1-IA, AG1-IB, AG22IIIB, AG3 Rhs1AP, AG3 123E, and AG8 WAC10335). To assess the repetitive element content of the newly sequenced *R. solani* AG1-IA isolates, RepeatScout [99] was used to predict de novo consensus repetitive element families in all the *R. solani* genomes used in this study. Through this approach, all low-complexity sequences, tandem repeats as well as repeat elements which contain less than 10 repeats sequences were filtered out. The resulting consensus repeat elements were then classified using TEclass [100] and mapped using RepeatMasker v4.0.7 [101].

### Comparative analyses and ortholog clustering

For pairwise genomic comparisons, MUMmer v3.23 [38] was used to align and compare the whole genome sequences of *R. solani* isolates. PROmer, a built-in MUMmer package that generates and aligns translations of all six reading frames for genome sequences of interest, was used to determine the extent of synteny between the genomes used in this study. OrthoFinder v 2.2.7 [102] was used for ortholog clustering to sort out single-copy gene families that would be the most phylogenetically informative. Single-copy ortholog genes in all fungal species were then aligned using ClustalW v2.1 [103], and poorly aligned regions were removed using trimAl v1.2 with the strict method [104]. RAxML v8.2.8 [105] and a bootstrap value of 1000 was used to construct a maximum likelihood-based phylogenetic tree. Ortholog genes were annotated with Gene Ontology (GO) annotation using Interproscan v5.20 [106].

### Gene family analyses

Genes encoding plant cell wall degrading enzymes were predicted and categorized using dbCAN HMMER v6 [107]. Each EC gene was collected from classification of CAZyDB-ec-info.txt.07-20-2017. Each classified group from dbCAN was subdivided using EC classification using BLAST 2.2.26. Aligning EC classified protein sequences using ClustalW 2.1 and removal of poorly aligned regions by trimAl v1.2 were preceded before phylogenetic analysis. Phylogeny trees were constructed using RAxML version 8.2.9 with a bootstrap value of 1000. We reconciled the gene tree resulting from this analysis with the species tree using NOTUNG 2.6 [108]. The secretome data of selected species were obtained from the Fungal Secretome Database (FSD) [109]. The database detects all possible secreted proteins by eliminating proteins with transmembrane or endoplasmic reticulum domains and using SignalP 3.0 [110]. The SSPs were then selected from each fungal secretome, considering proteins with a length shorter than 300 amino acids, as previously described [45]. Exonerate 2.4.0 was utilized to perform protein to genome sequence alignments of the effectors among *R. solani* genomes [111]. Genes encoding laccases and peroxidases were predicted using fPoxDB [112], while putative secondary metabolite biosynthesis gene clusters were identified using antiSMASH v3.0 [41], and the P450 database [113] was searched to predict cytochrome P450 genes in each genome. Transcription factors were identified using the Fungal Transcription Factor Database (FTFD) pipeline [114], which utilizes data from Interpro v12 [115].

### Statistical analysis

#### Chi-square tests of proportions

Chi-square tests of proportions for comparative analyses of CAZyme secondary metabolite biosynthesis clusters were performed using R [116].

## Supplementary Information

**Additional file 1: Figure S1.** Genome sequence assembly pipeline *R. solani* AG1-IA isolates B2, ADB, WGL and YN-7.

**Additional file 2: Table S1.** List of genomes of fungal species/genomes utilized in this study for comparative genome analyses (excluding the *R. solani* AG1-IA B2, ADB, WGL and YN-7).

**Additional file 3: Figure S2.** Synteny dot plots of *R. solani* genomes and reference B2 genome.

**Additional file 4: Table S2.** Pairwise genome alignments in terms of absolute and percent genome similarity among rice-infecting *R. solani* and different anastomosis groups (AG1-IB, AG2-2IIIB, AG3, AG8) using PROmer.

**Additional file 5: Table S3.** Absolute number and proportion of orthologs upon pairwise comparisons of 27 fungal genomes.

**Additional file 6: Table S4.** List of additional Basidiomycota genomes included for comparative protein-coding gene analysis.

**Additional file 7: Table S5.** Repetitive element profiles for *R. solani* AG1-IA, AG1-IB, AG2-2IIIB, AG3, and AG8 isolates.

**Additional file 8: Table S6.** The predicted number of protein-coding genes, secreted protein-coding genes, and small secreted protein-coding genes (SSPs) in 27 fungal genomes were used in this study.

**Additional file 9: Table S7.** List of conserved and unique SSPs found in all *R. solani* genomes used in this study.

**Additional file 10: Table S8.** Percentage of homology shared by 272 SSPs of *R. solani* B2 with *R. solani* AG1-IA (YN7, ADB, RNR), AG1-IB, AG2-2IIIB, AG3, and AG8.

**Additional file 11: Table S9.** Chi-square analysis of number of predicted Carbohydrate Active Enzymes (CAZymes) belonging carbohydrate-binding module (CBM), glycoside hydrolase (GH), carbohydrate esterase (CE), glycosyltransferase (GT), polysaccharide lyase (PL), and auxillary activity (AA) families observed in 11 fungal groupings based on lifestyle and host of genomes used in this study (*p* < 0.001).

**Additional file 12: Table S10.** Analysis of chi-square tests of selected CAZymes and secondary metabolites.

**Additional file 13: Table S11.** Chi-square analysis of the number of predicted lignocellulose, pectin, and monocot cell wall degrading genes observed across the 11 fungal groupings based on lifestyle and host of genomes used in this study (*p* < 0.001).

**Additional file 14: Figure S3.** Duplication-loss model of enriched EC gene families related to pectin modification in 27 fungal genomes utilized in this study.

**Additional file 15: Table S12.** Putative secondary metabolite biosynthesis gene clusters were observed in 11 fungal groupings based on lifestyle and host of genomes used in this study (*p* < 0.001).

## Data Availability

The genome assemblies of *R. solani* AG1-IA isolates B2, YN-7, ADB, and WGL generated during the current study are available in the NCBI repository, [accession numbers JACYCC000000000-JACYCF000000000 in PRJNA645424]. For fungal genomes used for comparative analyses in this study were downloaded from the NCBI, JGI and Broad Institute database; their accession numbers are listed in Additional file 2: Table S1 and Additional file 6: Table S4. The datasets supporting the conclusions of this article are included within the article and its additional files.

## Abbreviations

AG: Anastomosis group
ISG: Intraspecific group
ShB: Sheath blight
HG: Homogalacturonan
SMRT: Single Molecule Real-Time
CAZyme: Carbohydrate-active enzyme
AA: Auxiliary activity
MnP: Manganese peroxidase
VP: Versatile peroxidase
LPMO: Lytic polysaccharide monooxygenase
PKS: Polyketide synthase
TS: Terpene synthase
NRPS: Non-ribosomal peptide synthetase
SSP: Small secreted protein
GH: Glycoside hydrolase
CE: Carbohydrate esterase
GT: Glycosyltransferase
PL: Polysaccharide lyase
PG: Polygalacturonase
PME: Pectin methylesterase
PAE: Pectin acetylesterase
PNL: Pectin lyase

## References

[1] Miyake I (1910). Studien uber die Pilze der Reispflanze in Japan. J Coll Agric Imp Univ Tokyo.

[2] Hashiba T, Kobayashi T, Sneh B, Jabaji-Hare S, Neate S, Dijst G (1996). Rice diseases incited by *Rhizoctonia* species. *Rhizoctonia* species: taxonomy, molecular biology, ecology, pathology and disease control.

[3] Banniza S, Holderness M, Sreenivasaprasad S, Johnson R (2001). Rice sheath blight—pathogen biology and diversity. Major fungal diseases of rice.

[4] Willocquet L, Fernandez L, Savary SJPP (2000). Effect of various crop establishment methods practised by Asian farmers on epidemics of rice sheath blight caused by *Rhizoctonia solani*. Plant Pathol.

[5] Kim K-H, Cho J, Lee YH, Lee W-SJA (2015). Predicting potential epidemics of rice leaf blast and sheath blight in South Korea under the RCP 4.5 and RCP 8.5 climate change scenarios using a rice disease epidemiology model, EPIRICE. Agric For Meteorol.

[6] Ogoshi A (1987). Ecology and pathogenicity of anastomosis and intraspecific groups of *Rhizoctonia solani* Kühn. Annu Rev Phytopathol.

[7] Carling D, Baird R, Gitaitis R, Brainard K, Kuninaga S (2002). Characterization of AG-13, a newly reported anastomosis group of *Rhizoctonia solani*. Phytopathology..

[8] Ajayi-Oyetunde O, Bradley C (2018). *Rhizoctonia solani*: taxonomy, population biology and management of *Rhizoctonia* seedling disease of soybean. Plant Pathol.

[9] Gonzalez D, Rodriguez-Carres M, Boekhout T, Stalpers J, Kuramae EE, Nakatani AK (2016). Phylogenetic relationships of *Rhizoctonia* fungi within the Cantharellales. Fungal Biol.

[10] Priyatmojo A, Escopalao VE, Tangonan NG, Pascual CB, Suga H, Kageyama K, Hyakumachi M (2001). Characterization of a new subgroup of *Rhizoctonia solani* anastomosis group 1 (AG-1-ID), causal agent of a necrotic leaf spot on coffee. Phytopathology..

[11] Jones R, Belmar S (1989). Characterization and pathogenicity of *Rhizoctonia* spp. isolated from rice, soybean, and other crops grown in rotation with rice in Texas. Plant Dis.

[12] Pascual C, Toda T, Raymondo A, Hyakumachi M (2000). Characterization by conventional techniques and PCR of *Rhizoctonia solani* isolates causing banded leaf sheath blight in maize. Plant Pathol.

[13] Hane JK, Anderson JP, Williams AH, Sperschneider J, Singh KB (2014). Genome sequencing and comparative genomics of the broad host-range pathogen *Rhizoctonia solani* AG8. PLoS Genet.

[14] Ahuja S, Payak MJP (1982). Symptoms and signs of banded leaf and sheath blight of maize. Phytoparasitica..

[15] Giesler LJ, Yuen GY, Horst GLJP (1996). The microclimate in tall fescue turf as affected by canopy density and its influence on brown patch disease. Plant Dis.

[16] Zheng A, Lin R, Zhang D, Qin P, Xu L, Ai P, Ding L, Wang Y, Chen Y, Liu Y, Sun Z, Feng H, Liang X, Fu R, Tang C, Li Q, Zhang J, Xie Z, Deng Q, Li S, Wang S, Zhu J, Wang L, Liu H, Li P (2013). The evolution and pathogenic mechanisms of the rice sheath blight pathogen. Nat Commun.

[17] Wibberg D, Jelonek L, Rupp O, Hennig M, Eikmeyer F, Goesmann A, Hartmann A, Borriss R, Grosch R, Pühler A, Schlüter A (2013). Establishment and interpretation of the genome sequence of the phytopathogenic fungus *Rhizoctonia solani* AG1-IB isolate 7/3/14. J Biotechnol.

[18] Wibberg D, Rupp O, Jelonek L, Krober M, Verwaaijen B, Blom J (2015). Improved genome sequence of the phytopathogenic fungus *Rhizoctonia solani* AG1-IB 7/3/14 as established by deep mate-pair sequencing on the MiSeq (Illumina) system. J Biotechnol.

[19] Wibberg D, Andersson L, Tzelepis G, Rupp O, Blom J, Jelonek L, Pühler A, Fogelqvist J, Varrelmann M, Schlüter A, Dixelius C (2016). Genome analysis of the sugar beet pathogen *Rhizoctonia solani* AG2-2IIIB revealed high numbers in secreted proteins and cell wall degrading enzymes. BMC Genomics.

[20] Cubeta MA, Thomas E, Dean RA, Jabaji S, Neate SM, Tavantzis S (2014). Draft genome sequence of the plant-pathogenic soil fungus *Rhizoctonia solani* anastomosis group 3 strain Rhs1AP. Genome Announc.

[21] Wibberg D, Genzel F, Verwaaijen B, Blom J, Rupp O, Goesmann A, Zrenner R, Grosch R, Pühler A, Schlüter A (2017). Draft genome sequence of the potato pathogen *Rhizoctonia solani* AG3-PT isolate Ben3. Arch Microbiol.

[22] Patil VU, Girimalla V, Sagar V, Bhardwaj V, Chakrabarti SK (2017). Draft genome sequencing of *Rhizoctonia solani* anastomosis group 3 (AG3- PT) causing stem canker and black scurf of potato. Am J Potato Res.

[23] Losada L, Pakala SB, Fedorova ND, Joardar V, Shabalina SA, Hostetler J, Pakala SM, Zafar N, Thomas E, Rodriguez-Carres M, Dean R, Vilgalys R, Nierman WC, Cubeta MA (2014). Mobile elements and mitochondrial genome expansion in the soil fungus and potato pathogen *Rhizoctonia solani* AG-3. FEMS Microbiol Lett.

[24] Xia Y, Fei B, He J, Zhou M, Zhang D, Pan L, Li S, Liang Y, Wang L, Zhu J, Li P, Zheng A (2017). Transcriptome analysis reveals the host selection fitness mechanisms of the *Rhizoctonia solani* AG1IA pathogen. Sci Rep.

[25] Kwon YS, Kim SG, Chung WS, Bae H, Jeong SW, Shin SC, Jeong MJ, Park SC, Kwak YS, Bae DW, Lee YB (2014). Proteomic analysis of *Rhizoctonia solani* AG-1 sclerotia maturation. Fungal Biol..

[26] Anderson JP, Hane JK, Stoll T, Pain N, Hastie ML, Kaur P, Hoogland C, Gorman JJ, Singh KB (2016). Proteomic analysis of *Rhizoctonia solani* identifies infection-specific, redox associated proteins and insight into adaptation to different plant hosts. Mol Cell Proteomics.

[27] Lo Presti L, Lanver D, Schweizer G, Tanaka S, Liang L, Tollot M, Zuccaro A, Reissmann S, Kahmann R (2015). Fungal effectors and plant susceptibility. Annu Rev Plant Biol.

[28] Girard V, Dieryckx C, Job C, Job DJP (2013). Secretomes: the fungal strike force. Proteomics..

[29] Djamei A, Schipper K, Rabe F, Ghosh A, Vincon V, Kahnt J, Osorio S, Tohge T, Fernie AR, Feussner I, Feussner K, Meinicke P, Stierhof YD, Schwarz H, Macek B, Mann M, Kahmann R (2011). Metabolic priming by a secreted fungal effector. Nature..

[30] Khang CH, Berruyer R, Giraldo MC, Kankanala P, Park SY, Czymmek K, Kang S, Valent B (2010). Translocation of *Magnaporthe oryzae* effectors into rice cells and their subsequent cell-to-cell movement. Plant Cell.

[31] De Wit PJ, Mehrabi R, Van den Burg HA, Stergiopoulos I (2009). Fungal effector proteins: past, present and future. Mol Plant Pathol.

[32] Ciuffetti LM, Manning VA, Pandelova I, Betts MF, Martinez JP (2010). Host-selective toxins, Ptr ToxA and Ptr ToxB, as necrotrophic effectors in the *Pyrenophora tritici*-*repentis*-wheat interaction. New Phytol.

[33] Stergiopoulos I, Collemare J, Mehrabi R, De Wit PJGM (2013). Phytotoxic secondary metabolites and peptides produced by plant pathogenic Dothideomycete fungi. FEMS Microbiol Rev.

[34] Rytioja J, Hilden K, Yuzon J, Hatakka A, de Vries RP, Makela MR (2014). Plant-polysaccharide-degrading enzymes from Basidiomycetes. Microbiol Mol Biol Rev.

[35] Annis SL, Goodwin PH (1997). Recent advances in the molecular genetics of plant cell wall-degrading enzymes produced by plant pathogenic fungi. Eur J Plant Pathol.

[36] Cantarel BL, Coutinho PM, Rancurel C, Bernard T, Lombard V, Henrissat B. The carbohydrate-active EnZymes database (CAZy): an expert resource for Glycogenomics. Nucleic Acids Res 2009;37:D233–D238, Database, DOI: 10.1093/nar/gkn663. 10.1093/nar/gkn663 PMC2686590 18838391

[37] Lombard V, Golaconda Ramulu H, Drula E, Coutinho PM, Henrissat B (2014). The carbohydrate-active enzymes database (CAZy) in 2013. Nucleic Acids Res.

[38] Kurtz S, Phillippy A, Delcher AL, Smoot M, Shumway M, Antonescu C, Salzberg SL (2004). Versatile and open software for comparing large genomes. Genome Biol.

[39] Castanera R, Borgognone A, Pisabarro AG, Ramirez L (2017). Biology, dynamics, and applications of transposable elements in basidiomycete fungi. Appl Microbiol Biotechnol.

[40] Kidwell MGJG (2002). Transposable elements and the evolution of genome size in eukaryotes. Genetica..

[41] Weber T, Blin K, Duddela S, Krug D, Kim HU, Bruccoleri R, Lee SY, Fischbach MA, Müller R, Wohlleben W, Breitling R, Takano E, Medema MH (2015). antiSMASH 3.0-a comprehensive resource for the genome mining of biosynthetic gene clusters. Nucleic Acids Res.

[42] Rhoads A, Au KF (2015). PacBio sequencing and its applications. Genomics Proteomics Bioinform.

[43] Arakawa M, Inagaki K (2014). Molecular markers for genotyping anastomosis groups and understanding the population biology of *Rhizoctonia* species. J Gen Plant Pathol.

[44] Sharma M, Gupta S, Sharma TR (2005). Characterization of variability in *Rhizoctonia solani* by using morphological and molecular markers. J Phytopathol.

[45] Kim KT, Jeon J, Choi J, Cheong K, Song H, Choi G (2016). Kingdom-wide analysis of fungal small secreted proteins (SSPs) reveals their potential role in host association. Front Plant Sci.

[46] Anderson PK, Cunningham AA, Patel NG, Morales FJ, Epstein PR, Daszak P (2004). Emerging infectious diseases of plants: pathogen pollution, climate change and agrotechnology drivers. Trends Ecol Evol.

[47] Guyon K, Balagué C, Roby D, Raffaele S (2014). Secretome analysis reveals effector candidates associated with broad host range necrotrophy in the fungal plant pathogen *Sclerotinia sclerotiorum*. BMC Genomics.

[48] Gan P, Narusaka M, Kumakura N, Tsushima A, Takano Y, Narusaka Y, Shirasu K (2016). Genus-wide comparative genome analyses of *Colletotrichum* species reveal specific gene family losses and gains during adaptation to specific infection lifestyles. Genome Biol Evol.

[49] Pauly M, Keegstra K (2008). Cell-wall carbohydrates and their modification as a resource for biofuels. Plant J.

[50] Vogel J (2008). Unique aspects of the grass cell wall. Curr Opin Plant Biol.

[51] Carpita NC, Gibeaut DM (1993). Structural models of primary cell walls in flowering plants: consistency of molecular structure with the physical properties of the walls during growth. Plant J.

[52] Northcote D (1972). Chemistry of the plant cell wall. Annu Rev Plant Physiol.

[53] Zhong R, Ye ZH (2007). Regulation of cell wall biosynthesis. Curr Opin Plant Biol.

[54] Underwood W (2012). The plant cell wall: a dynamic barrier against pathogen invasion. Front Plant Sci.

[55] Bacete L, Mélida H, Miedes E, Molina A (2018). Plant cell wall-mediated immunity: cell wall changes trigger disease resistance responses. Plant J.

[56] Zhao Q, Dixon RA (2014). Altering the cell wall and its impact on plant disease: from forage to bioenergy. Annu Rev Phytopathol.

[57] Riley R, Salamov AA, Brown DW, Nagy LG, Floudas D, Held BW, Levasseur A, Lombard V, Morin E, Otillar R, Lindquist EA, Sun H, LaButti KM, Schmutz J, Jabbour D, Luo H, Baker SE, Pisabarro AG, Walton JD, Blanchette RA, Henrissat B, Martin F, Cullen D, Hibbett DS, Grigoriev IV (2014). Extensive sampling of basidiomycete genomes demonstrates inadequacy of the white-rot/brown-rot paradigm for wood decay fungi. Proc Natl Acad Sci.

[58] Kirk TK, Farrell RL (1987). Enzymatic" combustion": the microbial degradation of lignin. Annu Rev Microbiol.

[59] Hatakka A (1994). Lignin-modifying enzymes from selected white-rot fungi: production and role from in lignin degradation. FEMS Microbiol Rev.

[60] Makela MR, Donofrio N, de Vries RP (2014). Plant biomass degradation by fungi. Fungal Genet Biol.

[61] Eastwood DC, Floudas D, Binder M, Majcherczyk A, Schneider P, Aerts A, Asiegbu FO, Baker SE, Barry K, Bendiksby M, Blumentritt M, Coutinho PM, Cullen D, de Vries RP, Gathman A, Goodell B, Henrissat B, Ihrmark K, Kauserud H, Kohler A, LaButti K, Lapidus A, Lavin JL, Lee YH, Lindquist E, Lilly W, Lucas S, Morin E, Murat C, Oguiza JA, Park J, Pisabarro AG, Riley R, Rosling A, Salamov A, Schmidt O, Schmutz J, Skrede I, Stenlid J, Wiebenga A, Xie X, Kues U, Hibbett DS, Hoffmeister D, Hogberg N, Martin F, Grigoriev IV, Watkinson SC (2011). The plant cell wall–decomposing machinery underlies the functional diversity of forest fungi. Science..

[62] Blanchette RA (1991). Delignification by wood-decay fungi. Annu Rev Phytopathol.

[63] Worrall JJ, Anagnost SE, Zabel RA (1997). Comparison of wood decay among diverse lignicolous fungi. Mycologia..

[64] Yelle DJ, Ralph J, Lu F, Hammel KE (2008). Evidence for cleavage of lignin by a brown rot basidiomycete. Environ Microbiol.

[65] Hongo S, Sato K, Yokoyama R, Nishitani K (2012). Demethylesterification of the primary wall by PECTIN METHYLESTERASE35 provides mechanical support to the *Arabidopsis* stem. Plant Cell.

[66] Bouton S (2002). QUASIMODO1 encodes a putative membrane-bound glycosyltransferase required for normal pectin synthesis and cell adhesion in *Arabidopsis*. Plant Cell.

[67] Liu H, Ma Y, Chen N, Guo S, Liu H, Guo X (2014). Overexpression of stress-inducible OsBURP16, the β subunit of polygalacturonase 1, decreases pectin content and cell adhesion and increases abiotic stress sensitivity in rice. Plant Cell Environ.

[68] Levesque-Tremblay G, Pelloux J, Braybrook SA, Müller KJP (2015). Tuning of pectin methylesterification: consequences for cell wall biomechanics and development. Planta..

[69] O'Connell RJ, Thon MR, Hacquard S, Amyotte SG, Kleemann J, Torres MF, Damm U, Buiate EA, Epstein L, Alkan N, Altmüller J, Alvarado-Balderrama L, Bauser CA, Becker C, Birren BW, Chen Z, Choi J, Crouch JA, Duvick JP, Farman MA, Gan P, Heiman D, Henrissat B, Howard RJ, Kabbage M, Koch C, Kracher B, Kubo Y, Law AD, Lebrun MH, Lee YH, Miyara I, Moore N, Neumann U, Nordström K, Panaccione DG, Panstruga R, Place M, Proctor RH, Prusky D, Rech G, Reinhardt R, Rollins JA, Rounsley S, Schardl CL, Schwartz DC, Shenoy N, Shirasu K, Sikhakolli UR, Stüber K, Sukno SA, Sweigard JA, Takano Y, Takahara H, Trail F, van der Does HC, Voll LM, Will I, Young S, Zeng Q, Zhang J, Zhou S, Dickman MB, Schulze-Lefert P, ver Loren van Themaat E, Ma LJ, Vaillancourt LJ (2012). Lifestyle transitions in plant pathogenic Colletotrichum fungi deciphered by genome and transcriptome analyses. Nat Genet.

[70] Zhao Z, Liu H, Wang C, Xu JR (2013). Comparative analysis of fungal genomes reveals different plant cell wall degrading capacity in fungi. BMC Genomics.

[71] Mohnen D (2008). Pectin structure and biosynthesis. Curr Opin Plant Biol.

[72] Harholt J, Suttangkakul A, Vibe SH (2010). Biosynthesis of pectin. Plant Physiol.

[73] Wolf S, Mouille G, Pelloux J (2009). Homogalacturonan methyl-esterification and plant development. Mol Plant.

[74] Micheli F (2001). Pectin methylesterases: cell wall enzymes with important roles in plant physiology. Trends Plant Sci.

[75] Pelloux J, Rusterucci C, Mellerowicz EJ (2007). New insights into pectin methylesterase structure and function. Trends Plant Sci.

[76] Albersheim P, Darvill A, Roberts K, Sederoff R, Staehelin A (2011). Plant cell walls.

[77] Hocq L, Pelloux J, Lefebvre V (2017). Connecting homogalacturonan-type pectin remodeling to acid growth. Trends Plant Sci.

[78] Lespinet O, Wolf YI, Koonin EV, Aravind L (2002). The role of lineage-specific gene family expansion in the evolution of eukaryotes. Genome Res.

[79] Cooper RM, Longman D, Campbell A, Henry M, Lees PJP, Pathology MP (1988). Enzymic adaptation of cereal pathogens to the monocotyledonous primary wall. Physiol Mol Plant P.

[80] Zalewska-Sobczak J (1985). Sequential secretion of cell wall degrading enzymes by *Botrytis fabae* and *Fusarium avenaceum* during growth on host and non-host plants. Biochem Physiol Pflanz.

[81] King BC, Waxman KD, Nenni NV, Walker LP, Bergstrom GC, Gibson DM (2011). Arsenal of plant cell wall degrading enzymes reflects host preference among plant pathogenic fungi. Biotechnol Biofuels.

[82] Buanafina MM. Feruloylation in grasses: current and future perspectives. Mol Plant. 2009;2(5):861–72. 10.1093/mp/ssp067. 10.1093/mp/ssp067 19825663

[83] Vega-Sanchez ME, Verhertbruggen Y, Scheller HV, Ronald PC (2013). Abundance of mixed linkage glucan in mature tissues and secondary cell walls of grasses. Plant Signal Behav.

[84] Hatfield RD, Rancour DM, Marita JM (2016). Grass cell walls: a story of cross-linking. Front Plant Sci.

[85] Sumiyoshi M, Nakamura A, Nakamura H, Hakata M, Ichikawa H, Hirochika H, Ishii T, Satoh S, Iwai H (2013). Increase in cellulose accumulation and improvement of saccharification by overexpression of arabinofuranosidase in rice. PLoS One.

[86] Vega-Sanchez ME, Verhertbruggen Y, Christensen U, Chen X, Sharma V, Varanasi P (2012). Loss of cellulose synthase-like F6 function affects mixed-linkage glucan deposition, cell wall mechanical properties, and defense responses in vegetative tissues of rice. Plant Physiol.

[87] Collemare J, Lebrun MH. Fungal secondary metabolites: ancient toxins and novel effectors in plant–microbe interactions. In: Martin F, Kamoun S, editors. Effectors in plant–microbe interactions, vol. 2011. Chichester: Wiley; 2011. p. 377–400. 10.1002/9781119949138.ch15.

[88] Spanu PD, Abbott JC, Amselem J, Burgis TA, Soanes DM, Stüber K, Loren van Themaat EV, Brown JKM, Butcher SA, Gurr SJ, Lebrun MH, Ridout CJ, Schulze-Lefert P, Talbot NJ, Ahmadinejad N, Ametz C, Barton GR, Benjdia M, Bidzinski P, Bindschedler LV, Both M, Brewer MT, Cadle-Davidson L, Cadle-Davidson MM, Collemare J, Cramer R, Frenkel O, Godfrey D, Harriman J, Hoede C, King BC, Klages S, Kleemann J, Knoll D, Koti PS, Kreplak J, López-Ruiz FJ, Lu X, Maekawa T, Mahanil S, Micali C, Milgroom MG, Montana G, Noir S, O’Connell RJ, Oberhaensli S, Parlange F, Pedersen C, Quesneville H, Reinhardt R, Rott M, Sacristán S, Schmidt SM, Schön M, Skamnioti P, Sommer H, Stephens A, Takahara H, Thordal-Christensen H, Vigouroux M, Weßling R, Wicker T, Panstruga R (2010). Genome expansion and gene loss in powdery mildew fungi reveal tradeoffs in extreme parasitism. Science..

[89] Collemare J, Griffiths S, Iida Y, Jashni MK, Battaglia E, Cox RJ (2014). Secondary metabolism and biotrophic lifestyle in the tomato pathogen *Cladosporium fulvum*. PLoS One.

[90] Koren S, Walenz BP, Berlin K, Miller JR, Bergman NH, Phillippy AM (2017). Canu: scalable and accurate long-read assembly via adaptive k-mer weighting and repeat separation. Genome Res.

[91] Hunt M, De Silva N, Otto TD, Parkhill J, Keane JA, Harris SR (2015). Circlator: automated circularization of genome assemblies using long sequencing reads. Genome Biol.

[92] Pryszcz LP, Gabaldon T (2016). Redundans: an assembly pipeline for highly heterozygous genomes. Nucleic Acids Res.

[93] Walker BJ, Abeel T, Shea T, Priest M, Abouelliel A, Sakthikumar S, Cuomo CA, Zeng Q, Wortman J, Young SK, Earl AM (2014). Pilon: an integrated tool for comprehensive microbial variant detection and genome assembly improvement. PLoS One.

[94] Andrews S (2010). FastQC: a quality control tool for high throughput sequence data.

[95] Marçais G, Kingsford C (2011). A fast, lock-free approach for efficient parallel counting of occurrences of k-mers. Bioinformatics..

[96] Luo R, Liu B, Xie Y, Li Z, Huang W, Yuan J, He G, Chen Y, Pan Q, Liu Y, Tang J, Wu G, Zhang H, Shi Y, Liu Y, Yu C, Wang B, Lu Y, Han C, Cheung DW, Yiu SM, Peng S, Xiaoqian Z, Liu G, Liao X, Li Y, Yang H, Wang J, Lam TW, Wang J (2012). SOAPdenovo2: an empirically improved memory-efficient short-read de novo assembler. Gigascience..

[97] Zerbino DR, Birney E (2008). Velvet: algorithms for de novo short read assembly using de Bruijn graphs. Genome Res.

[98] Stanke M, Morgenstern B. AUGUSTUS: a web server for gene prediction in eukaryotes that allows user-defined constraints. Nucleic Acids Res 2005;33:W465-W4W7, Web Server, DOI: 10.1093/nar/gki458. 10.1093/nar/gki458 PMC1160219 15980513

[99] Price AL, Jones NC, Pevzner PA (2005). De novo identification of repeat families in large genomes. Bioinformatics..

[100] Abrusán G, Grundmann N, DeMester L, Makalowski W (2009). TEclass - a tool for automated classification of unknown eukaryotic transposable elements. Bioinformatics..

[101] Smit A, Hubley R, Green P. RepeatMasker Open-4.0. 2013. https://www.repeatmasker.org.

[102] Emms DM, Kelly S (2015). OrthoFinder: solving fundamental biases in whole genome comparisons dramatically improves orthogroup inference accuracy. Genome Biol.

[103] Larkin MA, Blackshields G, Brown N, Chenna R, McGettigan PA, McWilliam H (2007). Clustal W and Clustal X version 2.0. Bioinformatics..

[104] Capella-Gutiérrez S, Silla-Martínez JM, Gabaldón T (2009). TrimAl: a tool for automated alignment trimming in large-scale phylogenetic analyses. Bioinformatics.

[105] Stamatakis A (2014). RAxML version 8: a tool for phylogenetic analysis and post-analysis of large phylogenies. Bioinformatics..

[106] Finn RD, Attwood TK, Babbitt PC, Bateman A, Bork P, Bridge AJ (2016). InterPro in 2017—beyond protein family and domain annotations. Nucleic Acids Res.

[107] Huang L, Zhang H, Wu P, Entwistle S, Li X, Yohe T (2017). dbCAN-seq: a database of carbohydrate-active enzyme (CAZyme) sequence and annotation. Nucleic Acids Res.

[108] Chen K, Durand D, Farach-Colton M (2000). NOTUNG: a program for dating gene duplications and optimizing gene family trees. J Comput Biol.

[109] Choi J, Park J, Kim D, Jung K, Kang S, Lee Y-HJ (2010). Fungal secretome database: integrated platform for annotation of fungal secretomes. BMC Genomics.

[110] Bendtsen JD, Nielsen H, Von Heijne G, Brunak S (2004). Improved prediction of signal peptides: SignalP 3.0. J Mol Biol.

[111] Slater GS, Birney E (2005). Automated generation of heuristics for biological sequence comparison. BMC Bioinform.

[112] Choi J, Détry N, Kim K-T, Asiegbu FO, Valkonen JP, Lee Y-H (2014). fPoxDB: fungal peroxidase database for comparative genomics. BMC Microbiol.

[113] Park J, Lee S, Choi J, Ahn K, Park B, Park J, Kang S, Lee YH (2008). Fungal cytochrome P450 database. BMC Genomics.

[114] Park J, Park J, Jang S, Kim S, Kong S, Choi J, Ahn K, Kim J, Lee S, Kim S, Park B, Jung K, Kim S, Kang S, Lee YH (2008). FTFD: an informatics pipeline supporting phylogenomic analysis of fungal transcription factors. Bioinformatics..

[115] Hunter S, Apweiler R, Attwood TK, Bairoch A, Bateman A, Binns D (2008). InterPro: the integrative protein signature database. Nucleic Acids Res.

[116] R Core Team R (2013). R: a language and environment for statistical computing.

