# Correction to: Comparative genome analyses of four rice-infecting *Rhizoctonia solani* isolates reveal extensive enrichment of homogalacturonan modification genes

**DOI:** 10.1186/s12864-021-07631-0

**Published:** 2021-04-28

**Authors:** Da-Young Lee, Jongbum Jeon, Ki-Tae Kim, Kyeongchae Cheong, Hyeunjeong Song, Gobong Choi, Jaeho Ko, Stephen O. Opiyo, James C. Correll, Shimin Zuo, Sheshu Madhav, Guo-Liang Wang, Yong-Hwan Lee

**Affiliations:** 1 Department of Plant Pathology, The Ohio State University, Columbus, OH 43210 USA; 2 Fungal Bioinformatics Laboratory, Seoul National University, Seoul, 08826 South Korea; 3 Interdisciplinary Program in Agricultural Genomics, Seoul National University, Seoul, 08826 South Korea; 4 Department of Agricultural Life Science, Sunchon National University, Suncheon, 57922 South Korea; 5 Department of Agricultural Biotechnology, Seoul National University, Seoul, 08826 South Korea; 6 Ohio Agricultural Research and Development Center (OARDC) Molecular & Cellular Imaging Center (MCIC)-Columbus, The Ohio State University, Columbus, OH 43210 USA; 7 Department of Entomology & Plant Pathology, University of Arkansas, Fayetteville, AK 72701 USA; 8 Key Laboratory of Plant Functional Genomics of the Ministry of Education/ Jiangsu Key Laboratory of Crop Genomics and Molecular Breeding, Agricultural College of Yangzhou University, Yangzhou, China; 9 Indian Council of Agricultural Research-Indian Institute of Rice Research (ICAR-IIRR), Hyderabad, Telangana 500030 India; 10 Center for Fungal Genetic Resources, Plant Immunity Research Center, Plant Genomics and Breeding Institute, and Research Institute of Agriculture and Life Sciences, Seoul National University, Seoul, 08826 South Korea

**Correction to: BMC Genomics 22, 242 (2021)**

**https://doi.org/10.1186/s12864-021-07549-7**

Following publication of the original article [1], it was reported that there was an error in the affiliations of Yong-Hwan Lee. The given affiliations for Yong-Hwan Lee were 2, 3, 4, and 10, but should be 2, 3, 5, and 10. The original article has been corrected.
