# Supplementary figures and images for: Comparative genome analyses of four rice-infecting Rhizoctonia solani isolates reveal extensive enrichment of homogalacturonan modification genes
Source: BMC Genomics. 2021 Apr 7;22:242. doi: 10.1186/s12864-021-07549-7 (PMC8028249; doi:10.1186/s12864-021-07549-7)

**Figure S1.** Genome sequence assembly pipeline *R*. *solani* AG1-IA isolates B2, ADB, WGL and YN-7.


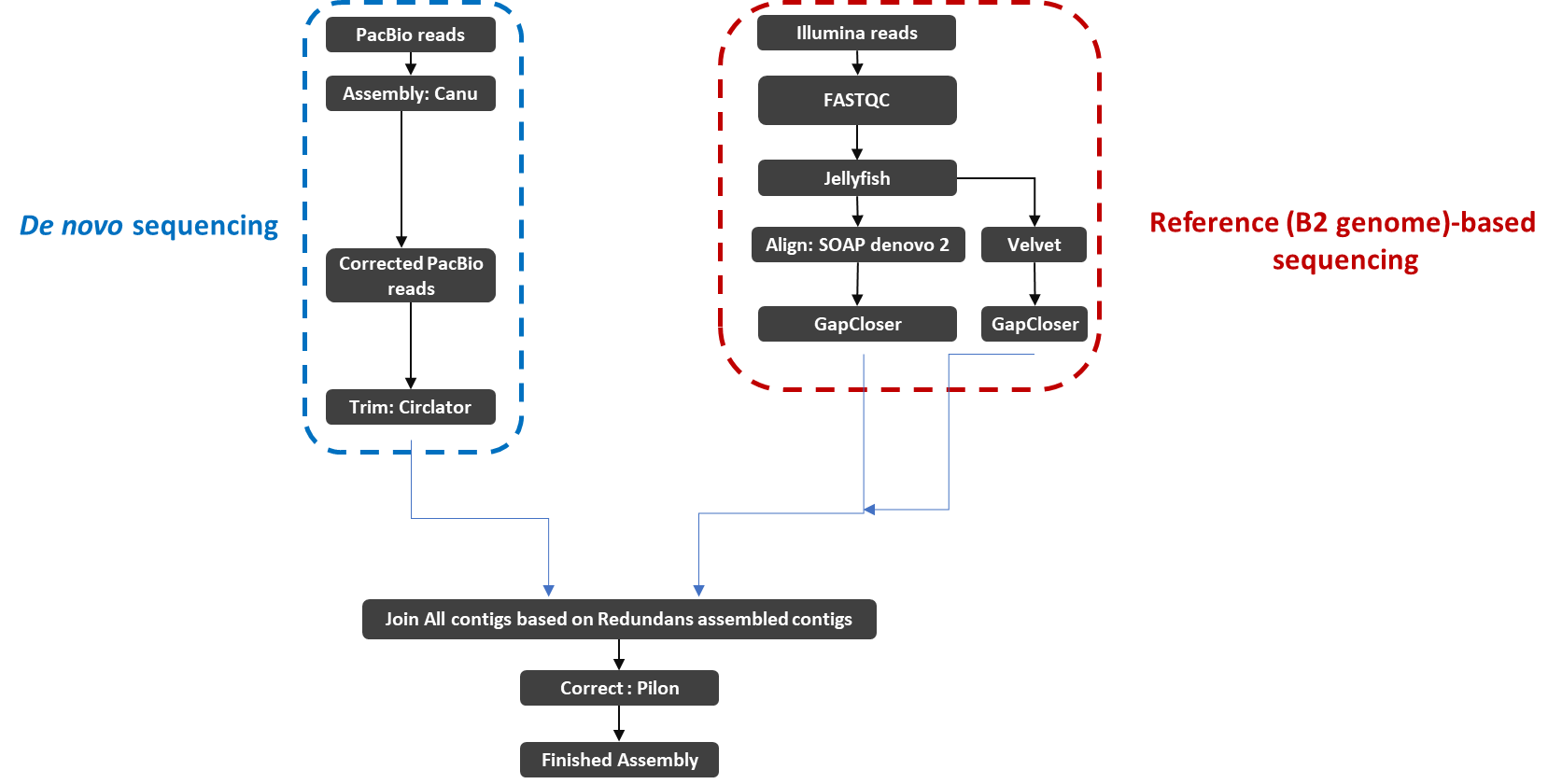

Supplement: Supplementary file 1 — Additional file 1: Figure S1. Genome sequence assembly pipeline R. solani AG1-IA isolates B2, ADB, WGL and YN-7. [file 12864_2021_7549_MOESM1_ESM.docx]
