# Supplementary material for: Comparative genome analyses of four rice-infecting Rhizoctonia solani isolates reveal extensive enrichment of homogalacturonan modification genes
Source: BMC Genomics. 2021 Apr 7;22:242. doi: 10.1186/s12864-021-07549-7 (PMC8028249; doi:10.1186/s12864-021-07549-7)
Supplement: Supplementary file 3 — Additional file 3: Figure S2. Synteny dot plots of R. solani genomes and reference B2 genome. [file 12864_2021_7549_MOESM3_ESM.docx]

**Figure S2.** Synteny dot plots of *R*. *solani* genomes and reference B2 genome.


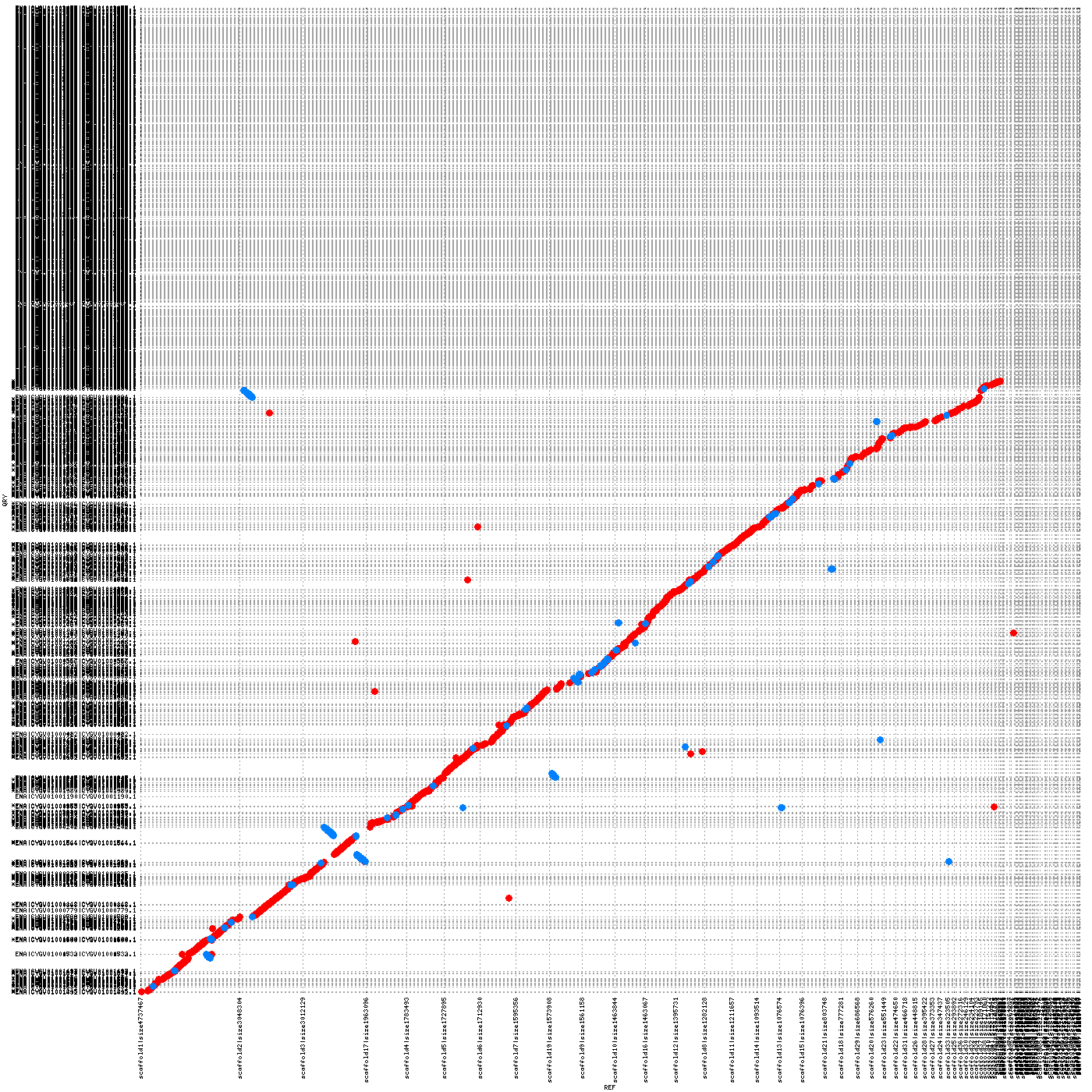

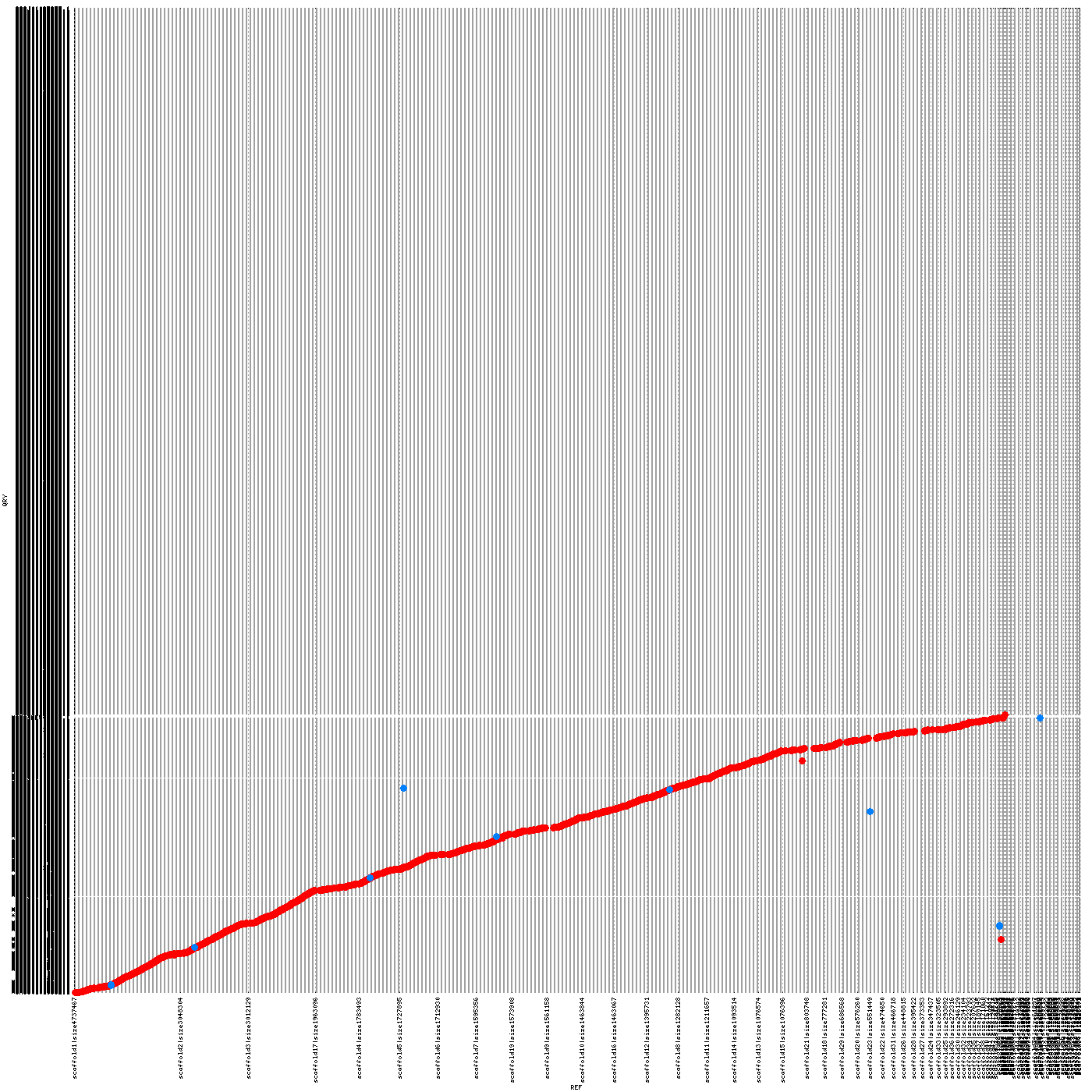

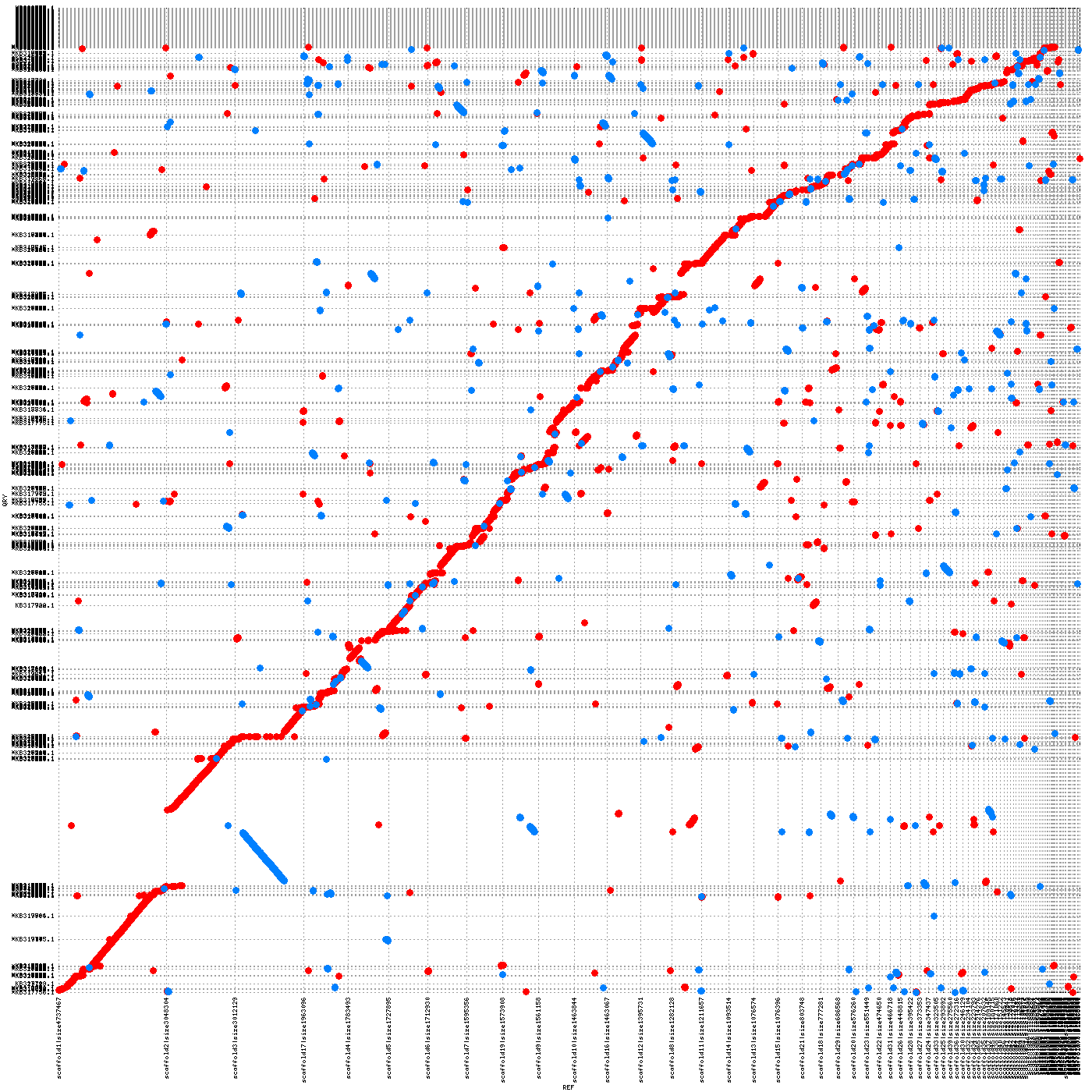

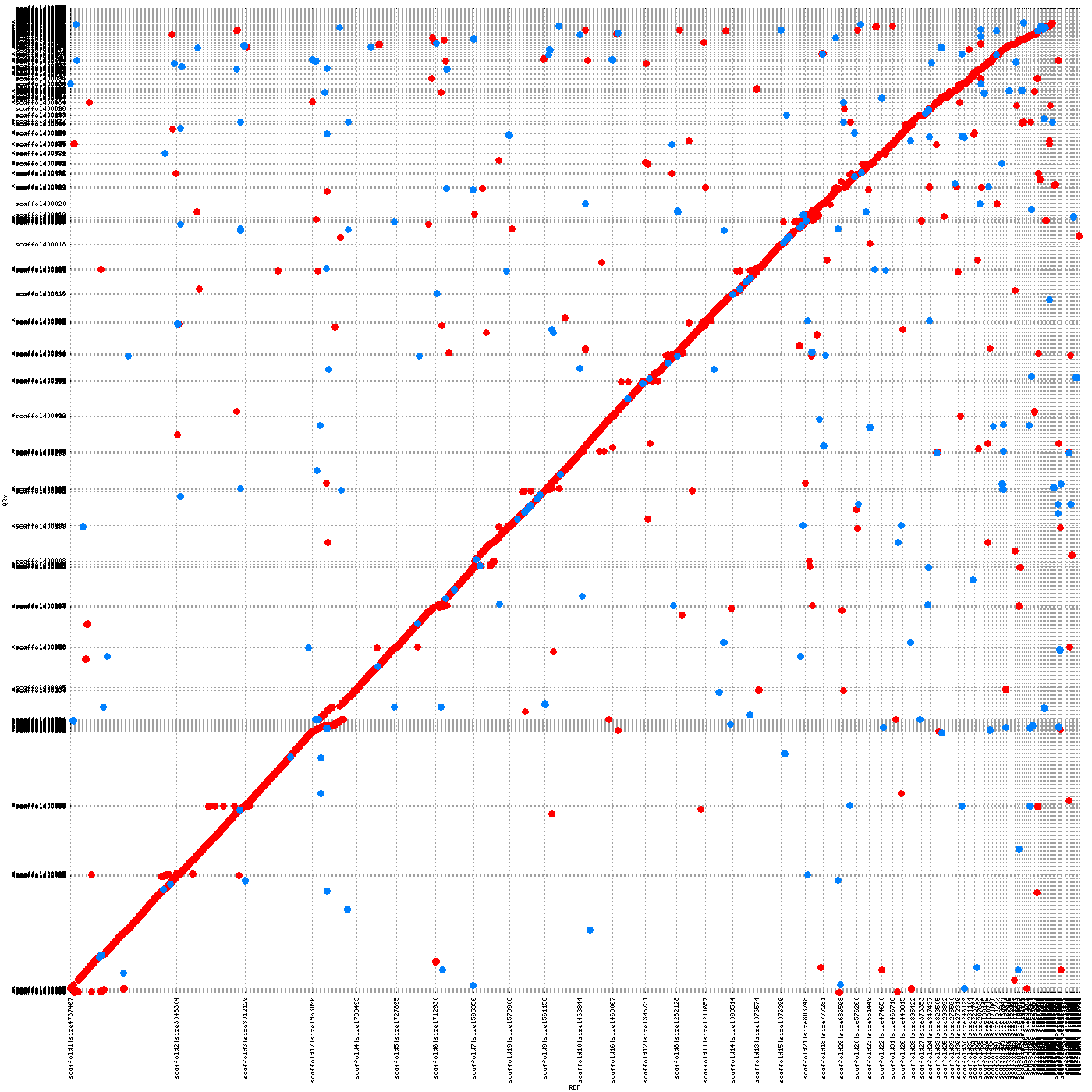


**AG1 IA YN-7**

**AG1 IA**

**AG1 IB**

**AG2**

**AG1-IA B2**

**AG1-IA B2**

**AG1-IA B2**

**AG1-IA B2**

**AG1-IA B2**

**AG1-IA B2**

**AG1-IA B2**

**AG1-IA B2**


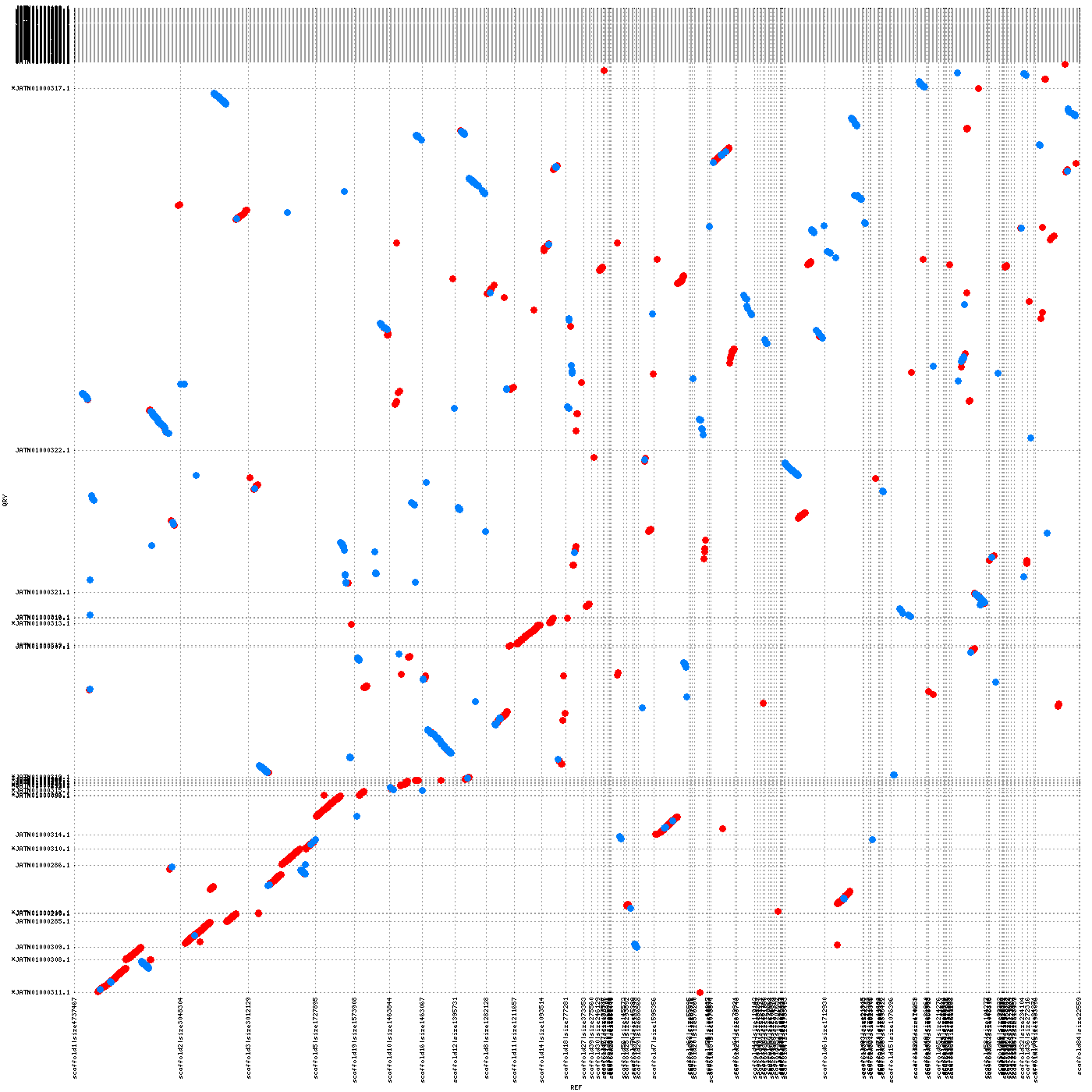

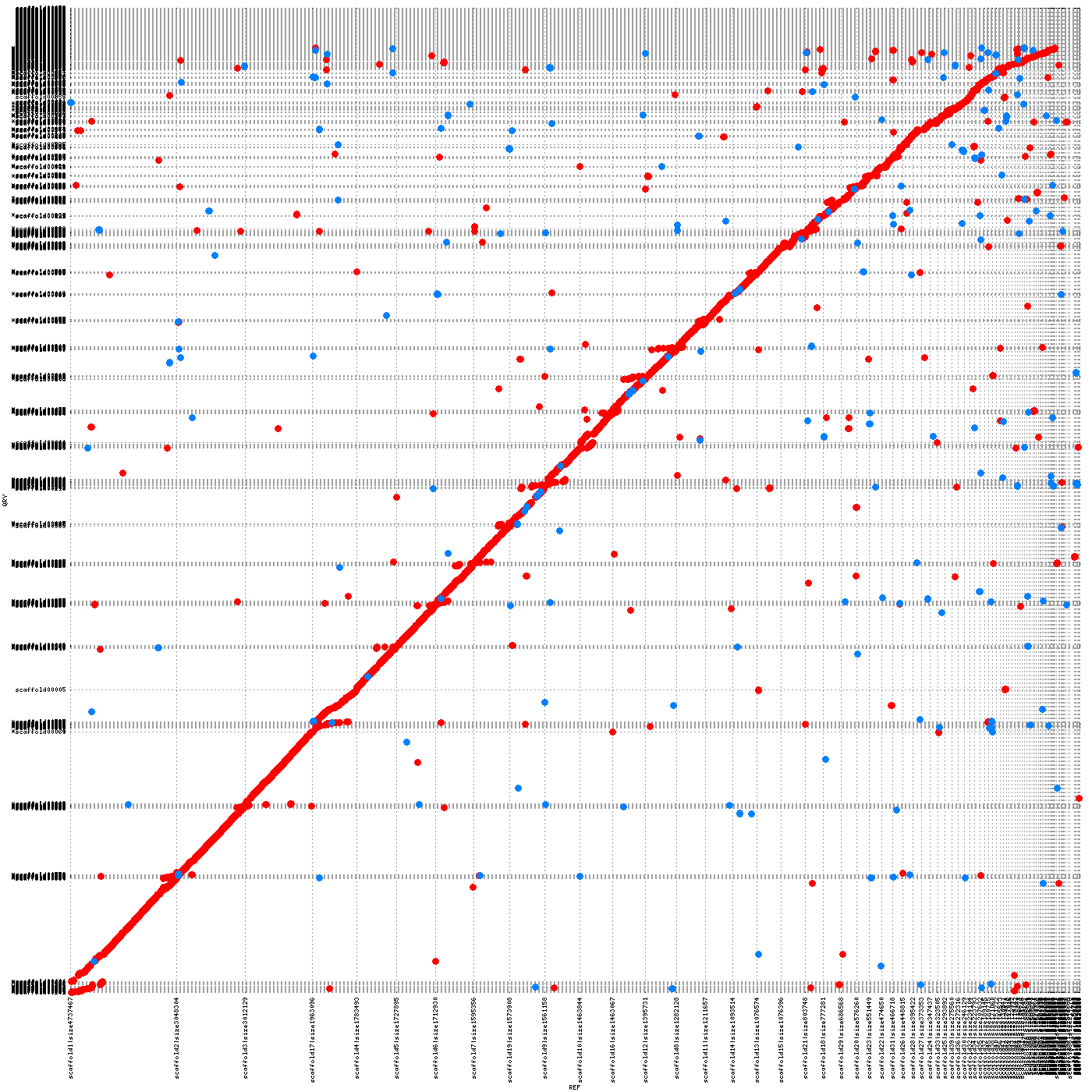

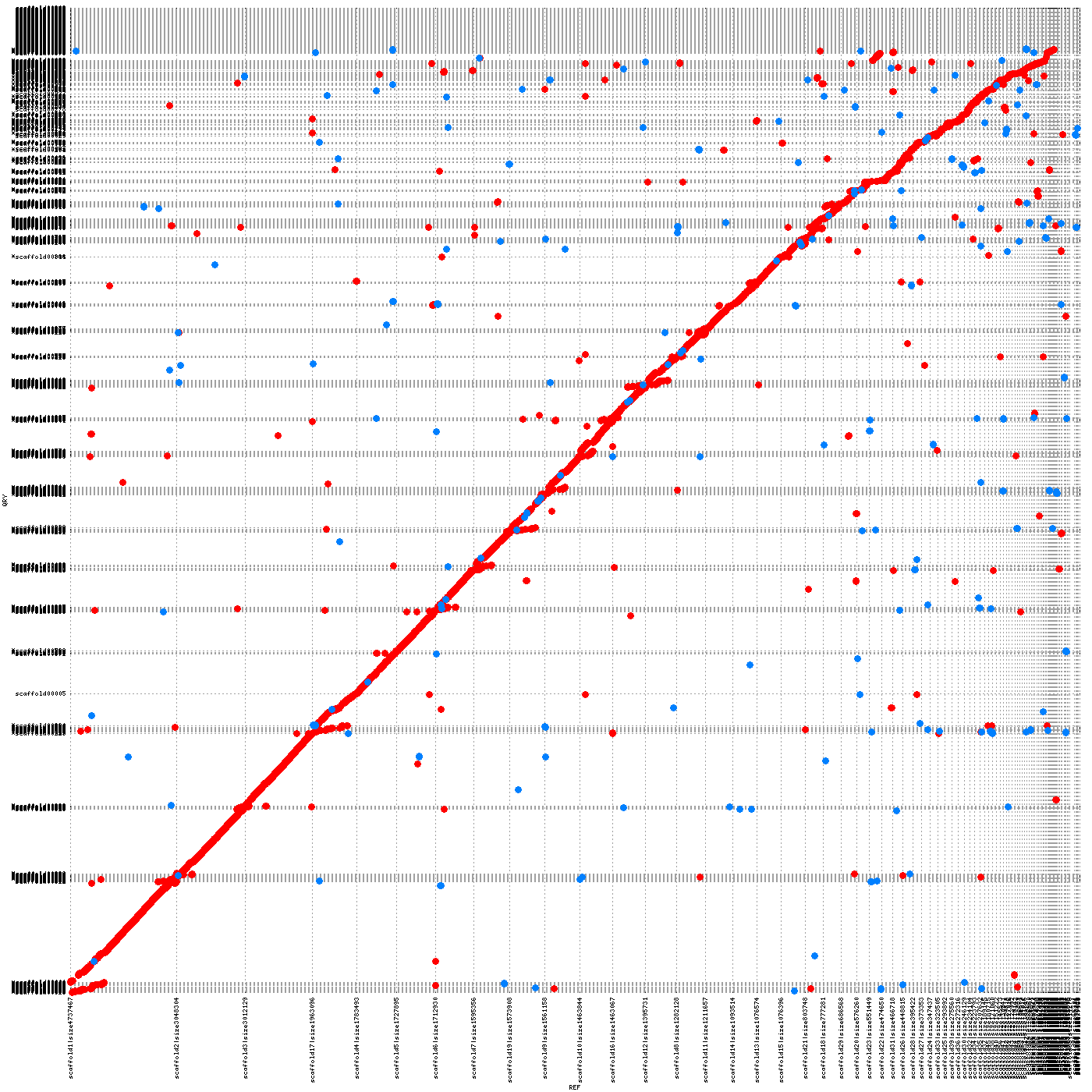

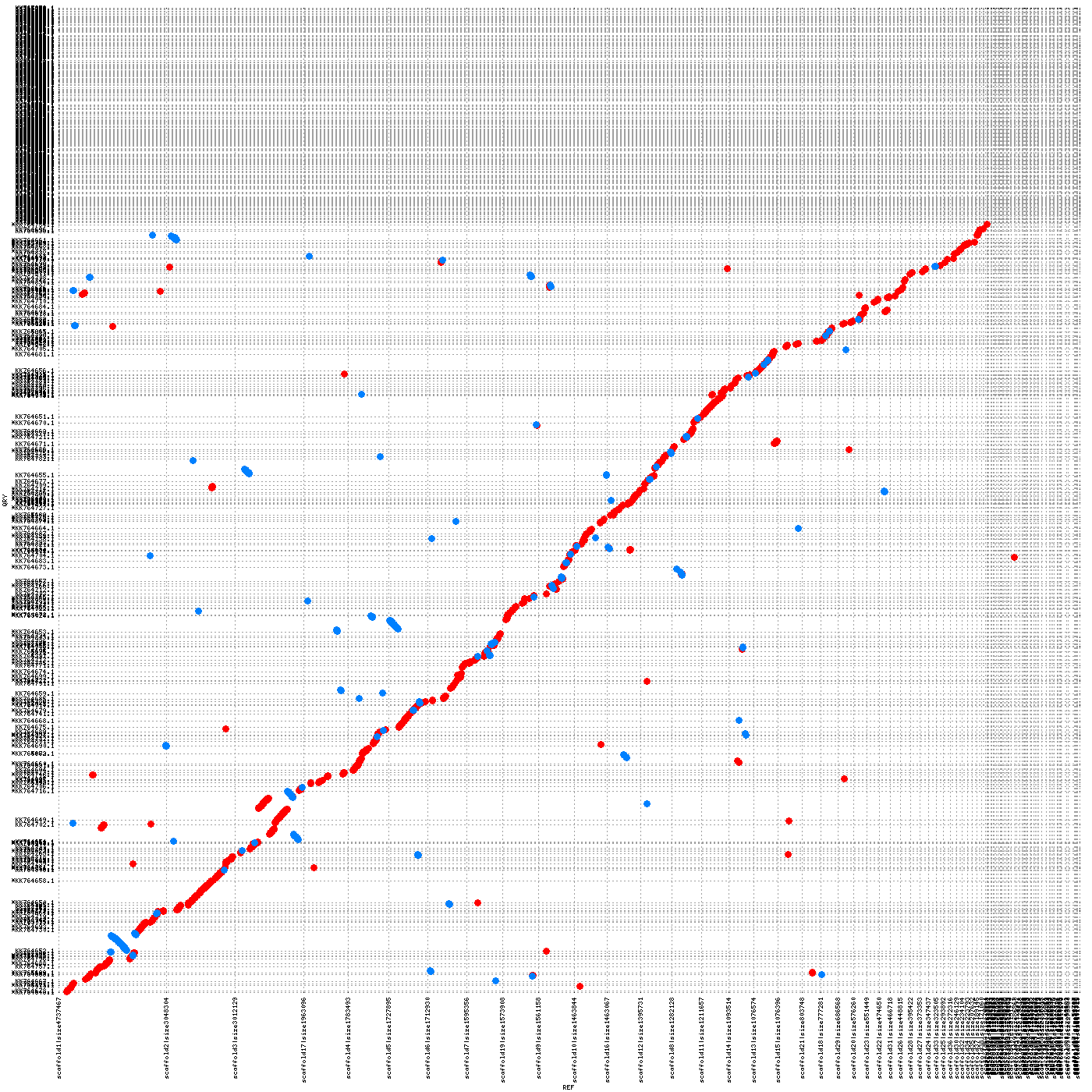


**AG8**

**AG3**

**AG1 IA WGL**

**AG1 IA ADB**

Synteny dot plots showing *R*. *solani* genomes aligned to the reference B2 genome. The x-axis of all the dot plots is the B2 genome and the y-axis denote the genome being compared.
