# Supplementary material for: Comparative genome analyses of four rice-infecting Rhizoctonia solani isolates reveal extensive enrichment of homogalacturonan modification genes
Source: BMC Genomics. 2021 Apr 7;22:242. doi: 10.1186/s12864-021-07549-7 (PMC8028249; doi:10.1186/s12864-021-07549-7)
Supplement: Supplementary file 4 — Additional file 4: Table S2. Pairwise genome alignments in terms of absolute and percent genome similarity among rice-infecting R. solani and different anastomosis groups (AG1-IB, AG2-2IIIB, AG3, AG8) using PROmer. [file 12864_2021_7549_MOESM4_ESM.docx]

**Table S2.** Pairwise genome alignments in terms of absolute and percent genome similarity among rice-infecting *R*. *solani* and other anastomosis groups (AG1-IB, AG2-2IIIB, AG3, AG8) using PROmer.

|  |  | | **Reference Genomes (Mb)** | | | | | | | | | | | |
| --- | --- | --- | --- | --- | --- | --- | --- | --- | --- | --- | --- | --- | --- | --- |
| **Query**  **Genomes** | **Genome size**  **(Mb)** | | **IA YN-7** | **IA B2** | **IA** | **AI ADB** | **AI WGL** | | **AG1-IB** | **AG2-2IIIB** | | **AG3** | **AG8** | |
| AG1-IA YN-7 | | 38.9 | - | 31.9 | 32.3 | 31.7 | 31.9 | 6.7 | | 4.7 | 4.7 | | | 2.5 |
| AG1-IA B2 | | 45 | 31.9 | - | 29.8 | 29.9 | 30.1 | 4.3 | | 2.9 | 2.8 | | | 1.5 |
| AG1-IA | | 36.9 | 32.3 | 29.7 | - | 30.8 | 30.9 | 6.4 | | 4.5 | 4.5 | | | 2.4 |
| AG1-IA ADB | | 39.9 | 31.7 | 29.9 | 30.8 | - | 38.2 | 6.6 | | 4.6 | 4.5 | | | 2.4 |
| AG1-IA WGL | | 40 | 31.9 | 30.1 | 30.9 | 38.2 | - | 6.6 | | 4.7 | 4.6 | | | 2.5 |
| AG1-IB | | 42.8 | 6.7 | 4.3 | 6.4 | 6.6 | 6.6 | - | | 3.7 | 3.8 | | | 2 |
| AG2-2IIIB | | 56 | 4.7 | 2.9 | 4.5 | 4.7 | 4.7 | 3.7 | | - | 3.8 | | | 2 |
| AG3 | | 51.7 | 4.7 | 2.8 | 4.5 | 4.6 | 4.6 | 3.8 | | 3.8 | - | | | 5.4 |
| AG8 | | 39.8 | 2.5 | 1.5 | 2.4 | 2.4 | 2.5 | 2 | | 2 | 5.4 | | | - |

|  | **Percentage Similarity (%)** | | | | | | | | |
| --- | --- | --- | --- | --- | --- | --- | --- | --- | --- |
| **Query**  **Genomes** | **IA YN-7** | **IA B2** | **IA** | **IA ADB** | **IA WGL** | **AG1-IB** | **AG2-2IIIB** | **AG3** | **AG8** |
| AG1-IA YN-7 | - | 70.9 | 87.5 | 79.4 | 79.8 | 15.7 | 8.4 | 9.1 | 6.3 |
| AG1-IA B2 | 82 | - | 80.8 | 74.9 | 75.3 | 10 | 5.2 | 5.4 | 3.8 |
| AG1-IA | 83 | 66 | - | 77.2 | 77.3 | 15 | 8 | 8.7 | 6 |
| AG1-IA ADB | 81.5 | 66.4 | 83.5 | - | 95.5 | 15.4 | 8.2 | 8.7 | 6 |
| AG1-IA WGL | 82 | 66.9 | 83.7 | 95.7 | - | 15.4 | 8.4 | 8.9 | 6.3 |
| AG1-IB | 17.2 | 9.6 | 17.3 | 16.5 | 16.5 | - | 6.6 | 7.4 | 5 |
| AG2-2IIIB | 12.1 | 6.4 | 12.2 | 11.8 | 11.8 | 8.6 | - | 7.4 | 5 |
| AG3 | 12.1 | 6.2 | 12.2 | 11.5 | 11.5 | 8.9 | 6.8 | - | 13.6 |
| AG8 | 6.4 | 3.3 | 6.5 | 6 | 6.3 | 4.7 | 3.6 | 10.4 | - |
