# Supplementary material for: Comparative genome analyses of four rice-infecting Rhizoctonia solani isolates reveal extensive enrichment of homogalacturonan modification genes
Source: BMC Genomics. 2021 Apr 7;22:242. doi: 10.1186/s12864-021-07549-7 (PMC8028249; doi:10.1186/s12864-021-07549-7)
Supplement: Supplementary file 6 — Additional file 6: Table S4. List of additional Basidiomycota genomes included for comparative protein-coding gene analysis. [file 12864_2021_7549_MOESM6_ESM.docx]

Table S4. List of additional Basidiomycota genomes included for comparative protein-coding gene analysis.

| **Phylum** | **Fungal species/AG** | **Isolate** | **GenBank/RefSeq accession** | **Database** |
| --- | --- | --- | --- | --- |
| Basidiomycota | *Ustilago_bromivora* | UB2112 | GCA_900080155.1 | NCBI |
|  | *Ustilago_hordei* | Uh4857-4 | GCA_000286035.1 | NCBI |
|  | *Ustilago_maydis* | 521 | GCF_000328475.2 | NCBI |
|  | *Ustilago_trichophora* | NBRC100155 | GCA_900323505.1 | NCBI |
|  | *Trametes_cinnabarina* | BRFM137 | GCA_000765035.1 | NCBI |
|  | *Trametes_coccinea* | BRFM310 | GCA_002092935.1 | NCBI |
|  | *Trametes_pubescens* | FBCC735 | GCA_001895945.1 | NCBI |
|  | *Trametes_versicolor* | FP-101664 SS1 | GCF_000271585.1 | NCBI |
