# Supplementary material for: Comparative genome analyses of four rice-infecting Rhizoctonia solani isolates reveal extensive enrichment of homogalacturonan modification genes
Source: BMC Genomics. 2021 Apr 7;22:242. doi: 10.1186/s12864-021-07549-7 (PMC8028249; doi:10.1186/s12864-021-07549-7)
Supplement: Supplementary file 8 — Additional file 8: Table S6. The predicted number of protein-coding genes, secreted protein-coding genes, and small secreted protein-coding genes (SSPs) in 27 fungal genomes were used in this study. [file 12864_2021_7549_MOESM8_ESM.docx]

**Table S6.** Predicted number of protein-coding genes, secreted protein-coding genes and small secreted protein-coding genes in (SSPs) 27 fungal genomes used in this study.

| **Fungal species/AG** | **Protein-coding genes** | **Secreted protein-coding genes** | **Small secreted protein-coding genes (SSPs)** |
| --- | --- | --- | --- |
| *Rhizoctonia* *solani* AG1-1A YN-7 | 9,715 | 887 | 270 |
| *Rhizoctonia* *solani* AG1-1A B2 | 11,505 | 818 | 272 |
| *Rhizoctonia* *solani* AG1-1A | 10,039 | 835 | 279 |
| *Rhizoctonia* *solani* AG1-1A ADB | 10,010 | 888 | 263 |
| *Rhizoctonia* *solani* AG1-1A WGL | 10,044 | 883 | 268 |
| *Rhizoctonia* *solani* AG1-1B | 12,616 | 832 | 460 |
| *Rhizoctonia* *solani* AG2-2IIIB | 11,897 | 1,047 | 349 |
| *Rhizoctonia* *solani* AG3 Rhs1AP | 12,726 | 999 | 369 |
| *Rhizoctonia* *solani* AG8 WAC10335 | 13,952 | 753 | 417 |
| *Botryobasidium* *botryosum* | 16,526 | 941 | 493 |
| *Piriformospora indica* | 11,767 | 692 | 317 |
| *Pleurotus* *ostreatus* | 12,330 | 869 | 368 |
| *Armillaria* *ostoyae* | 22,299 | 1,183 | 528 |
| *Heterobasidion* *irregulare* | 13,405 | 627 | 253 |
| *Postia* *placenta* | 12,541 | 582 | 232 |
| *Dacryopinax* sp. | 10,242 | 523 | 237 |
| *Ustilago* *maydis* | 6,783 | 485 | 222 |
| *Puccinia* *graminis* f. sp. *tritici* | 15,979 | 1,382 | 993 |
| *Trichoderma* *virens* | 12,427 | 818 | 321 |
| *Fusarium* *graminearum* | 13,321 | 963 | 379 |
| *Verticillium* *dahlia* | 10,575 | 854 | 297 |
| *Magnaporthe* *oryzae* | 12,991 | 1,452 | 817 |
| *Neurospora* *crassa* | 9,935 | 648 | 237 |
| *Blumeria* *graminis* f. sp. *hordei* | 6,495 | 498 | 341 |
| *Stagonospora* *nodorum* | 15,983 | 1,108 | 560 |
| *Cochliobolus* *miyabeanus* | 12,007 | 1,013 | 427 |
| *Pyrenophora* *tritici*-*repentis* | 12,169 | 942 | 437 |
