# Supplementary material for: Comparative genome analyses of four rice-infecting Rhizoctonia solani isolates reveal extensive enrichment of homogalacturonan modification genes
Source: BMC Genomics. 2021 Apr 7;22:242. doi: 10.1186/s12864-021-07549-7 (PMC8028249; doi:10.1186/s12864-021-07549-7)
Supplement: Supplementary file 11 — Additional file 11: Table S9. Chi-square analysis of number of predicted Carbohydrate Active Enzymes (CAZymes) belonging carbohydrate-binding module (CBM), glycoside hydrolase (GH), carbohydrate esterase (CE), glycosyltransferase (GT), polysaccharide lyase (PL), and auxillary activity (AA) families observed in 11 fungal groupings based on lifestyle and host of genomes used in this study (p < 0.001). [file 12864_2021_7549_MOESM11_ESM.docx]

**Table S9.** Chi-square analysis of number of predicted Carbohydrate Active Enzymes (CAZymes) belonging carbohydrate-binding module (CBM), glycoside hydrolase (GH), carbohydrate esterase (CE), glycosyltransferase (GT), polysaccharide lyase (PL) and auxillary activity (AA) families observed in 11 fungal groupings based on lifestyle and host of genomes used in this study (p < 0.001).

|  | **CAZyme family** | | | | | | |
| --- | --- | --- | --- | --- | --- | --- | --- |
| **Groupings based on lifestyle and hosts** | **CBM** | **CE** | **GH** | **GT*** | **PL** | **AA** | **TOTAL** |
| *R. solani* AG1-IA | 87 | 110 | 292 | 86 | 82 | 121 | 725 |
| Other *R*. *solani* AGs | 88 | 110 | 282 | 77 | 76 | 131 | 718 |
| Unclassified wood rot | 65 | 83 | 188 | 65 | 12 | 117 | 491 |
| White rot | 66 | 104 | 242 | 73 | 22 | 131 | 612 |
| Brown rot | 26 | 76 | 157 | 82 | 7 | 38 | 376 |
| Necrotroph (Cereals) | 55 | 122 | 263 | 97 | 15 | 128 | 646 |
| Hemibiotroph | 80 | 126 | 267 | 99 | 7 | 116 | 674 |
| Necrotroph (Dicot) | 63 | 106 | 269 | 93 | 38 | 104 | 623 |
| Biotroph | 11 | 52 | 117 | 70 | 2 | 23 | 270 |
| Symbiont | 93 | 93 | 240 | 82 | 10 | 77 | 535 |
| Saprotroph | 51 | 67 | 198 | 88 | 4 | 73 | 443 |
| Null | 62 | 95 | 229 | 83 | 25 | 96 | 556 |

*chi-square failed to reject null hypothesis

Null hypothesis: All grouping based on lifestyle and hosts have equal number of a particular CAZyme family genes.

| **Groups** | **Fungal species** |
| --- | --- |
| *R. solani* AG1-IA | *Rhizoctonia* *solani* AG1-IA YN-7 |
|  | *Rhizoctonia* *solani* AG1-IA B2 |
|  | *Rhizoctonia* *solani* AG1-IA Zheng |
|  | *Rhizoctonia* *solani* AG1-IA ADB |
|  | *Rhizoctonia* *solani* AG1-IA WGL |
| Other *R. solani* | *Rhizoctonia* *solani* AG1-IB |
|  | *Rhizoctonia* *solani* AG2-2IIIB |
|  | *Rhizoctonia* *solani* AG3 |
|  | *Rhizoctonia* *solani* AG8 |
| White rot | *Pleurotus* *ostreatus* |
|  | *Armillaria ostoyae* |
|  | *Heterobasidion irregulare* |
| Brown rot | *Postia placenta* |
|  | *Dacryopinax* sp. |
| Unclassified wood rot | *Botryobasidium botryosum* |
| Necrotroph (Cereal) | *Stagonospora nodorum* |
|  | *Cochliobolus miyabeanus* |
|  | *Pyrenophora tritici-repentis* |
|  | *Fusarium graminearum* |
| Biotroph | *Ustilago maydis* |
|  | *Puccinia graminis* |
|  | *Blumeria graminis* |
| Symbiont | *Piriformospora indica* |
|  | *Trichoderma virens* |
| Hemibiotroph | *Magnaporthe oryzae* |
| Saproptroph | *Neurospora crassa* |
| Necrotroph (Dicot) | *Verticillium dahliae* |
