# Supplementary material for: Comparative genome analyses of four rice-infecting Rhizoctonia solani isolates reveal extensive enrichment of homogalacturonan modification genes
Source: BMC Genomics. 2021 Apr 7;22:242. doi: 10.1186/s12864-021-07549-7 (PMC8028249; doi:10.1186/s12864-021-07549-7)
Supplement: Supplementary file 14 — Additional file 14: Figure S3. Duplication-loss model of enriched EC gene families related to pectin modification in 27 fungal genomes utilized in this study. [file 12864_2021_7549_MOESM14_ESM.docx]

**Figure S3.** Duplication-loss model of enriched EC gene families related to pectin modification in 27 fungal genomes utilized in this study.

-5

-1

-

**
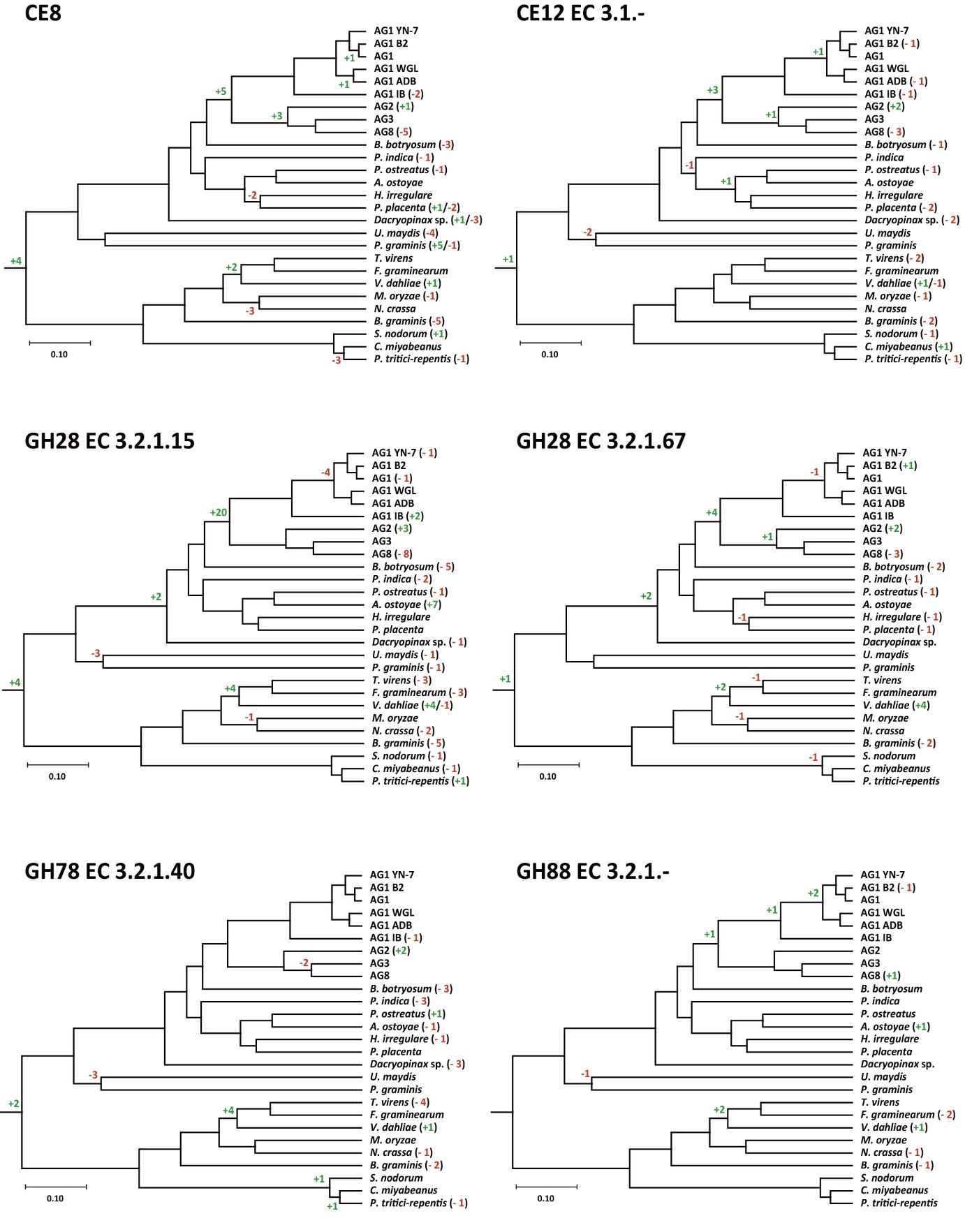
**


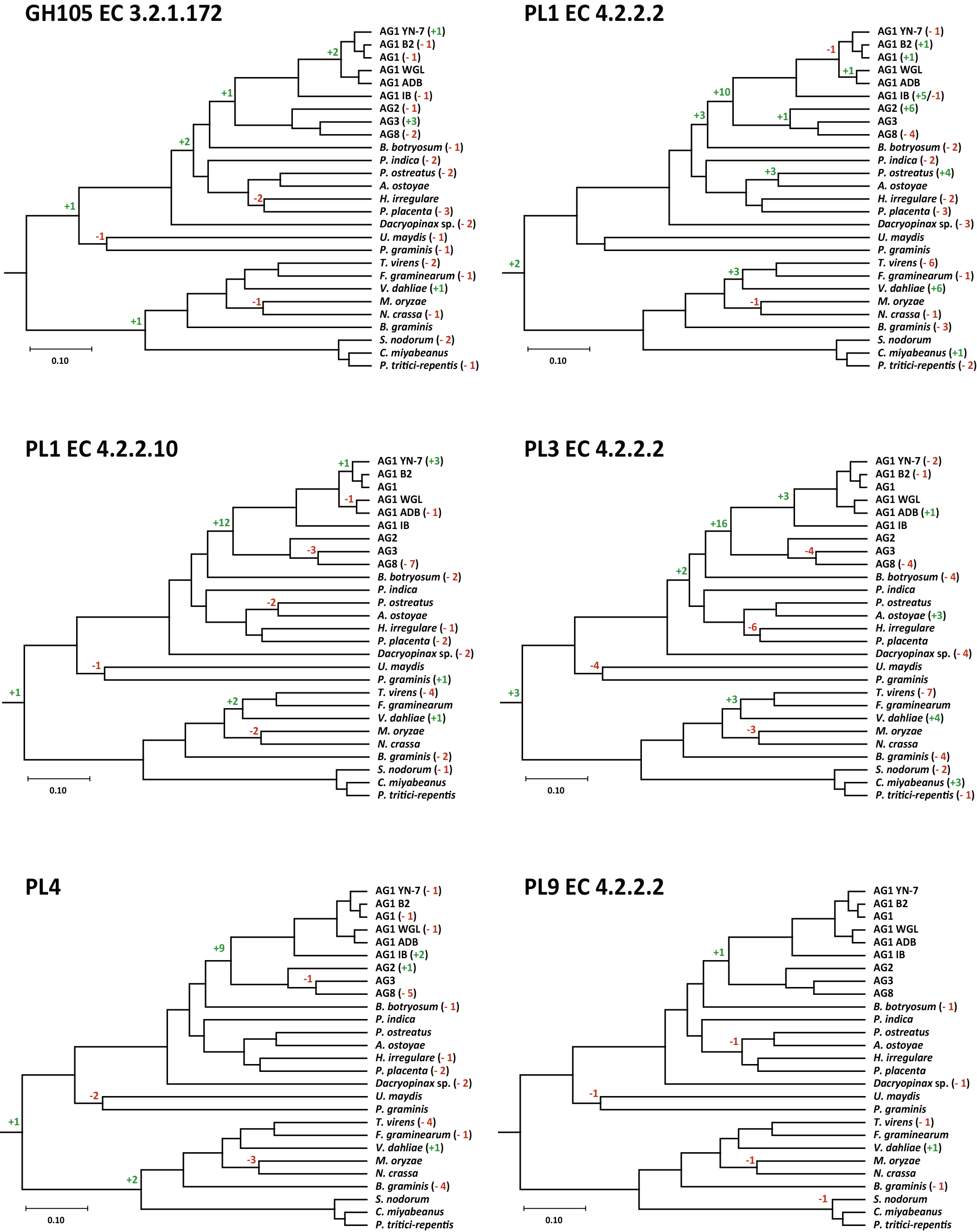


Duplication-loss model of EC subgroups in PL, PG, PME. Green number indicate duplication of gene(s) and Red number indicate loss of gene(s)
