# Supplementary material for: Comparative genome analyses of four rice-infecting Rhizoctonia solani isolates reveal extensive enrichment of homogalacturonan modification genes
Source: BMC Genomics. 2021 Apr 7;22:242. doi: 10.1186/s12864-021-07549-7 (PMC8028249; doi:10.1186/s12864-021-07549-7)
Supplement: Supplementary file 15 — Additional file 15: Table S12. Putative secondary metabolite biosynthesis gene clusters were observed in 11 fungal groupings based on lifestyle and host of genomes used in this study (p < 0.001). [file 12864_2021_7549_MOESM15_ESM.docx]

**Table S12.** Putative secondary metabolite biosynthesis gene clusters observed in 11 fungal groupings based on lifestyle and host of genomes used in this study (p < 0.001).

|  | **Type 1 PKS** | **Type 3 PKS*** | **NRPS** | **Terpenes*** | **DMATs*** | **Linaridin*** |
| --- | --- | --- | --- | --- | --- | --- |
| *R*. *solani* AG1 IA | 0 | 0 | 1 | 9 | 0 | 1 |
| Other *R*. *solani* AGs | 0 | 0 | 2 | 8 | 0 | 0 |
| Unclassified wood rot | 5 | 0 | 4 | 6 | 0 | 0 |
| White rot | 3 | 0 | 1 | 13 | 0 | 0 |
| Brown rot | 4 | 0 | 0 | 10 | 1 | 0 |
| Necrotroph (Cereal) | 17 | 1 | 11 | 8 | 1 | 0 |
| Hemibiotroph | 25 | 2 | 15 | 15 | 3 | 0 |
| Necrotroph (Dicot) | 9 | 1 | 4 | 5 | 0 | 0 |
| Biotroph | 1 | 0 | 2 | 3 | 0 | 0 |
| Symbiont | 12 | 0 | 13 | 7 | 1 | 0 |
| Saprotroph | 7 | 1 | 2 | 4 | 1 | 1 |
| Null | 7 | 1 | 5 | 8 | 1 | 0 |

*chi-square failed to reject null hypothesis

Null hypothesis: All grouping based on lifestyle and hosts have equal number of a particular secondary metabolite biosynthesis gene clusters.
